# Multi-parametric analyses to investigate dependencies of normal left atrial strain by cardiovascular magnetic resonance feature tracking

**DOI:** 10.1038/s41598-022-16143-0

**Published:** 2022-07-18

**Authors:** Jan Eckstein, Hermann Körperich, Lech Paluszkiewicz, Wolfgang Burchert, Misagh Piran

**Affiliations:** 1 Institute for Radiology, Nuclear Medicine and Molecular Imaging, Heart and Diabetes Centre North-Rhine Westphalia, Ruhr-University of Bochum, Georgstr. 11, 32545 Bad Oeynhausen, Germany; 2 Clinic for Thoracic and Cardiovascular Surgery, Heart and Diabetes Centre North-Rhine Westphalia, Ruhr-University of Bochum, Bad Oeynhausen, Germany

**Keywords:** Cardiology, Medical research

## Abstract

Left-atrial (LA) strain is the result of complex hemodynamics, which may be better characterized using a multiparametric approach. Cardiovascular magnetic resonance (CMR) feature tracking was used to perform a comprehensive LA strain assessment of 183 enrolled healthy volunteers (11–70 years, 97 females, median 32.9 ± 28.3 years). Novel strain dependencies were assessed using multi-parametric regression (MPR) analyses. LA volumetric data, left ventricular strain, transmitral and pulmonary venous blood flow parameters were utilized to create clusters for MPR of all subjects and a heart rate controlled subgroup (pulse: 60–75/min, N = 106). The LA reservoir(r) and conduit(c) strains of the total cohort were significantly elevated (p ≤ 0.001) in women (r: 49.7 ± 12.9%, c: 32.0 ± 11.0%) compared to men (r: 42.9 ± 11.4%, c: 26.1 IQ 10.5%). In contrast, there were no gender-specific differences (p > 0.05) for subgroup LA reservoir, conduit and booster(b) strains (all, r: 47.3 ± 12.7%; c: 29.0 IQ 15.5%; b: 17.6 ± 5.4%) and strain rates (all, 2.1 IQ 1.0 s^−1^; − 2.9 IQ 1.5 s^−1^; − 2.3 IQ 1.0 s^−1^). MPR found large effect sizes (|R^2^|≥ 0.26) for correlations between strain and various cardiac functional parameters. Largest effect size was found for the association between LA conduit strain and LA indexed booster volume, LA total ejection fraction, left ventricular global radial strain and E-wave (|R^2^|= 0.437). In addition to providing normal values for sex-dependent LA strain and strain rate, no gender differences were found with modified heart rate. MPR analyses of LA strain/strain rate and various cardiac functional parameters revealed that heart rate control improved goodness-of-fit for the overall model.

## Introduction

Myocardial strain studies the deformation between the contractile and relaxed state of the heart. Similar to the ejection fraction (EF), strain represents a load-dependent estimation of cardiac function. However, strain additionally acknowledges the spatial dimensions of contractile function in the form of longitudinal, radial and circumferential strain^1^. In particular, left atrial strain is a valuable diagnostic measure for the diagnosis and progression of cardiovascular diseases such as hypertension, atrial fibrillation, heart failure and cardiac amyloidosis^2–6^. It represents the shortening between the superior left atrial wall and the annular ring of the mitral valve and can be divided into three physiological phases. The reservoir phase occurs during ventricular systole until the opening of the mitral valve, when blood from pulmonary venous return accumulates in the left atrium. The subsequent conduit phase continues after mitral valve opening until the onset of left atrial contraction. The final booster phase begins with the contraction of the left atrium until the end of the ventricular diastole and leads to the active filling of the left ventricle^7,8^.

Speckle tracking and tissue doppler imaging are useful and widely available clinical tools for quantifying left atrial strain^9,10^. However, notable limitations of echocardiography include dependence on operator experience, reduced reproducibility, through-plane motion, as well as its patient echogenicity. Furthermore, the left atrium is characterized by complex geometric aspects with its thin interatrial septum, the pulmonary veins and the left atrial appendage. To overcome the geometric obstacles and limitations of echocardiography, cardiac chamber quantification by cardiovascular magnetic resonance (CMR) imaging has set the gold standard^11–13^.

Until today, CMR-quantified sex- and age-dependent left atrial strain of healthy individuals remain data limited^13,14^. In addition, the influence of heart rate on left atrial strain has not been studied so far. This study contributes CMR-attained normal left atrial strain values and investigates the effect of heart rate on left atrial strain using a large cohort of healthy participants. Moreover, we aimed to provide novel insights utilizing multiparametric analyses of the left atrial strain to better characterize the complexity of strain mechanics.

## Results

### Baseline characteristics of study cohort

This study entailed 183 healthy subjects ages 11–70 years (97 females and 86 males, median ± interquartile range 32.9 IQ 28.3 years of age). There was no gender difference for median age. The mean left ventricular ejection fraction (LV-EF) of all subjects was 65% ± 5%. BSA-indexed muscle mass (MM_i_) and BSA-indexed volumetric parameters were greater in males compared to females (p < 0.001). The baseline characteristics are summarized in Table 1.

**Table 1:** Baseline characteristics of healthy participants.

	All (n = 183)	Women (n = 97)	Men (n = 86)	p^†^
**All heart rates**				
Age (years)	32.9 {28.3}^a^ (11–70)	33.8 {25.9}^a^ (11–68)	32.7 {30.0}^a^ (13–70)	0.629
HR (bpm)	67 {14}^a^ (47–111)	70 {13}^a^ (47–111)	65 {14}^a^ (47–100)	0.026
Weight (kg)	69 {21}^a^ (38–120)	61 {11}^a^ (38–93)	81 ± 14 (50–120)	< 0.001
Height (cm)	173 ± 11 (140–200)	166 {11}^a^ (140–180)	182 ± 8 (161–200)	< 0.001
BSA (m^2^)	1.8 ± 0.2 (1.2–2.6)	1.7 ± 0.2 (1.2–2.0)	2.0 ± 0.2 (1.5–2.6)	< 0.001
BMI (kg/m^2^)	23.2 {4.9}^a^ (17–35)	22.3 {4.0}^a^ (16.7–34.6)	24.6 ± 3.5 (17.2–33.2)	< 0.001
LV-EDV_i_ (ml/m^2^)	76 ± 10 (48–103)	72 ± 9 (48–97)	80 ± 9 (55–103)	< 0.001
LV-ESV_i_ (ml/m^2^)	26 ± 5 (14–43)	24 ± 5 (14–40)	29 ± 5 (18–43)	< 0.001
LV-SV_i_ (ml/m^2^)	49 ± 7 (31–66)	48 ± 6 (31–64)	51 ± 6 (37–66)	< 0.001
LV-EF (%)	65 ± 5 (54–77)	66 ± 5 (57–76)	64 ± 5 (54–77)	0.003
MM_i_ (g/m^2^)	56 {15}^a^ (37–81)	50 {11}^a^ (37–73)	64 ± 7 (50–81)	< 0.001

Normally distributed values were expressed as mean ± standard deviation, otherwise as median (^a^) with interquartile range. Ranges in parentheses. ^†^p-value related to gender. Normally distributed data were tested by the unpaired t-test, otherwise with the Mann–Whitney-U-test. *BSA* body surface area, *BMI* body mass index, *LV-EDV*_*i*_ BSA-indexed end-diastolic volume, *LV-ESV*_*i*_ BSA-indexed end-systolic volume, *LV-SV*_*i*_ BSA-indexed stroke volume, *LV-EF* left ventricular ejection fraction, *MM*_*i*_ BSA-indexed muscle-mass, *HR* heart rate.

### Left atrial strains and volumes of the total cohort and heart rate specific subgroup

In the total cohort, females had higher left atrial strain rates, reservoir strain and conduit strain compared to males (Table 2). No gender difference was observed for left atrial booster strain (p = 0.058). In contrast, in the subgroup with a selected heart rate between 60 and 75 beats per minute (bpm), neither strain nor strain rate differed statistically significantly between the two cohorts at all stages of the cardiac (Table 3). Furthermore, heart rate selection diminished gender differences in left atrial chamber volume indices and ejection fraction phases. Thereby gender differences for indexed minimum left atrial volume (LA-Vol_min_i_) and passive left atrial ejection fraction (LA-EF_con_) became statistically insignificant after heart rate selection.

**Table 2:** Left atrial strain and volumes of the total cohort.

	All (n = 183)	Women (n = 97)	Men (n = 86)	p†
**Left atrial function**				
LA-Vol_max_i_ (ml/m^2^)	46.6 {12.4}^a^ (27.3–77.6)	45.4 {12.0}^a^ (30.6–74.0)	50.3 ± 9.6 (27.3–77.6)	0.002
LA-Vol_min_i_ (ml/m^2^)	20.7 {6.7}^a^ (10.3–39.8)	20.0 {5.8}^a^ (10.5–39.3)	21.8 {8.0}^a^ (10.3–39.8)	0.003
LA-Vol_boo_i_ (ml/m^2^)	32.5 {11.9}^a^ (15.0–58.1)	29.4 {9.8}^a^ (17.4–52.3)	36.0 ± 9.1 (15.0–58.1)	< 0.001
LA-EF_total_ (%)	55 ± 5 (40–67)	56 ± 5 (40–67)	55 ± 6 (41–65)	0.127
LA-EF_con_ (%)	31 ± 8 (13–54)	33 ± 8 (13–54)	29 ± 7 (15–48)	0.001
LA-EF_boo_ (%)	35 {9}^a^ (5–49)	34 {9}^a^ (5–46)	35 ± 6 (19–49)	0.065
**Left atrial strain**				
LA-S_res_ (%)	44.7 {16.7}^a^ (19.3–81.1)	49.7 ± 12.9 (24.1–81.1)	42.9 ± 11.4 (19.3–75.7)	< 0.001
LA-S_con_ (%)	28.6 {14.7}^a^ (8.3–61.8)	32.0 ± 11.0 (8.3–61.8)	26.1 {10.5}^a^ (10.7–59.2)	0.001
LA-S_boo_ (%)	16.9 {6.9}^a^ (4.2–35.0)	17.7 ± 5.4 (4.7–35.0)	16.1 ± 5.3 (4.2–33.0)	0.058
LA-SR_res_ (s^−1^)	2.1 {1.0}^a^ (0.6–5.0)	2.2 {1.1}^a^ (0.6–5.0)	2.0 {0.8}^a^ (0.9–4.1)	0.028
LA-SR_con_ (s^−1^)	− 2.9 {1.6}^a^ (− 8.9 to 3.9)	− 3.2 {1.9}^a^ (− 8.9 to 3.9)	− 2.8 {1.2}^a^ (− 6.2 to 2.6)	0.019
LA-SR_boo_ (s^−1^)	− 2.2 {1.1}^a^ (− 5.5 to 0.0)	− 2.4 {1.0}^a^ (− 5.5 to − 0.9)	− 2.0 {1.1}^a^ (− 4.5 to 0.0)	0.032

Normally distributed values were expressed as mean ± standard deviation, otherwise as median (^a^) with interquartile range. Ranges in parentheses. ^†^p-value related to gender. Normally distributed data were tested by the unpaired t-test, otherwise with the Mann–Whitney-U-test. *LA-Vol*_*max_i*_ indexed maximum left atrial volume, *LA-Vol*_*min_i*_ indexed minimum left atrial volume, *LA-Vol*_*boo_i*_ indexed booster left atrial volume, *LA-EF*_*total*_ total left atrial ejection fraction, *LA-EF*_*con*_ passive left atrial ejection fraction, *LA-EF*_*boo*_ booster left atrial ejection fraction, *LA-S*_*res*_ reservoir left atrial strain, *LA-S*_*con*_ conduit left atrial strain, *LA-S*_*boo*_ booster left atrial strain, *LA-SR*_*res*_ reservoir left atrial strain rate, *LA-SR*_*con*_ conduit left atrial strain rate, *LA-SR*_*boo*_ booster left atrial strain rate.

**Table 3:** Left atrial strain and volumes of the subgroup with heart rates between 60 to 75 beats-per-minute.

	All (106)	Women (60)	Men (46)	p^†^
HR (bpm)	68 {8}^a^	69 {8}^a^	68 {9}^a^	0.202
**Left atrial function**				
LA-Vol_max_i_ (ml/m^2^)	47.3 ± 8.4 (27.3–77.6)	45.9 ± 8.0 (32.1–67.4)	49.2 ± 8.7 (27.3–77.6)	0.049
LA-Vol_min_i_ (ml/m^2^)	20.7 {6.6}^a^ (10.3–39.3)	20.2 {6.3}^a^ (13.3–39.3)	21.9 ± 5.2 (10.3–37.7)	0.207
LA-Vol_boo_i_ (ml/m^2^)	33.2 ± 7.6 (15.0–55.7)	30.7 {11.7}^a^ (22.0–52.3)	34.8 ± 7.8 (15.0–55.7)	0.047
LA-EF_total_ (%)	56 {7}^a^ (40–65)	55 {6}^a^ (40–62)	56 ± 5 (41–65)	0.489
LA-EF_con_ (%)	30 ± 7 (13–48)	31 ± 7 (13–46)	30 ± 7 (19–48)	0.442
LA-EF_boo_ (%)	36 ± 6 (22–49)	35 ± 5 (23–46)	37 ± 6 (22–49)	0.060
**Left atrial strain**				
LA-S_res_ (%)^#^	47.3 ± 12.7 (19.3–81.1)	49.2 ± 13.2 (24.1–81.1)	44.9 ± 11.8 (19.3–75.7)	0.079
LA-S_con_ (%)^#^	29.0 {15.5}^a^ (8.3–59.2)	31.1 ± 11.3 (8.3–58.4)	27.0 {13.0}^a^ (10.7–59.2)	0.109
LA-S_boo_ (%)^#^	17.6 ± 5.4 (4.8–35.0)	18.1 ± 6.0 (4.8–35.0)	17.0 ± 4.6 (7.2–29.3)	0.297
LA-SR_res_ (s^−1^)^#^	2.1 {1.0}^a^ (0.6–5.0)	2.2 {1.0}^a^ (0.6–5.0)	1.9 {0.9}^a^ (0.9–3.4)	0.278
LA-SR_con_ (s^−1^)^#^	− 2.9 {1.5}^a^ (− 7.2 to 3.9)	− 3.0 {1.7}^a^ (− 7.2 to 3.9)	− 2.8 {1.0}^a^ (− 6.2 to − 1.6)	0.389
LA-SR_boo_ (s^−1^)^#^	− 2.3 {1.0}^a^ (− 4.9 to − 0.1)	− 2.4 {1.0}^a^ (− 4.9 to − 0.9)	− 2.2 {1.0}^a^ (− 4.5 to − 0.1)	0.483

Normally distributed values were expressed as mean ± standard deviation, otherwise as median (^a^) with interquartile range. Ranges in parentheses. ^†^p-value related to gender. ^#^No statistical difference between the total cohort (see Table 2) and the heart rate subgroup. Normally distributed data were tested by the unpaired t-test, otherwise with the Mann–Whitney-U-test. *LA-Vol*_*max_i*_ indexed maximum left atrial volume, *LA-Vol*_*min_i*_ indexed minimum left atrial volume, *LA-Vol*_*boo_i*_ indexed booster left atrial volume, *LA-EF*_*total*_ total left atrial ejection fraction, *LA-EF*_*con*_ passive left atrial ejection fraction, *LA-EF*_*boo*_ booster left atrial ejection fraction, *LA-S*_*res*_ reservoir left atrial strain, *LA-S*_*con*_ conduit left atrial strain, *LA-S*_*boo*_ booster left atrial strain, *LA-SR*_*res*_ reservoir left atrial strain rate, *LA-SR*_*con*_ conduit left atrial strain rate, *LA-SR*_*boo*_ booster left atrial strain rate.

### Cardiovascular functional parameters

All mean global left ventricular strain values were higher in females than in males (p < 0.001, Table 4). The statistical significance did not change when heart rate was limited to 60–75 bpm (p < 0.001, Table 5). There were no gender differences in the other parameters when all subjects were considered, except for the E-wave (E), which was greater in females. Heart rate selection was associated with a greater E/e′ ratio in females and a greater systolic excursion of the mitral annulus (MAPSE) in males. All other parameters remained comparable between both genders after heart rate selection.

**Table 4:** Cardiovascular functional parameter of the total cohort.

	All	Women	Men	p†
**All heart rates**				
LV-GLS (%)	− 16.9 ± 1.7 (− 22.3 to − 12.9)	− 17.6 ± 1.6 (− 22.3 to − 14.2)	− 16.1 ± 1.5 (− 20.8 to − 12.9)	< 0.001
LV-GCS (%)	− 19.2 ± 2.0 (− 24.8 to − 14.1)	− 20.1 ± 1.8 (− 24.8 to − 15.6)	− 18.2 ± 1.8 (− 21.8 to − 14.1)	< 0.001
LV-GRS (%)	33.6 {8.0}^a^ (21.4–53.9)	36.5 {6.9}^a^ (24.0–53.9)	31.3 ± 4.9 (21.4–42.1)	< 0.001
S/D	1.12 {0.57}^a^ (0.43–2.61)	1.09 {0.54}^a^ (0.53–2.61)	1.14 {0.63}^a^ (0.43–2.47)	0.847
E/A	1.86 {0.85}^a^ (0.81–5.14)	1.86 {0.84}^a^ (0.81–5.14)	1.91 ± 0.62 (0.82–3.74)	0.352
E (cm/s)	52.9 ± 9.1 (25.4–76.2)	54.5 ± 8.8 (25.4–76.2)	51.0 ± 9.0 (31.9–72.6)	0.010
A (cm/s)	28.3 {11.0}^a^ (14.8–57.5)	28.5 {9.9}^a^ (14.8–54.0)	27.5 {11.7}^a^ (15.0–57.5)	0.824
é (cm/s)	13.3 ± 4.0 (4.2–23.2)	13.3 ± 3.8 (4.2–23.2)	13.3 ± 4.2 (4.8–23.0)	0.988
E/é	4.16 {1.56}^a^ (1.73–13.28)	4.22 {1.42}^a^ (1.73–13.28)	3.99 {1.47}^a^ (2.00–9.26)	0.137
MAPSE (cm)	1.7 ± 0.3 (0.9–2.5)	1.6 ± 0.3 (1.0–2.5)	1.7 ± 0.3 (0.9–2.4)	0.196
SI	1.26 {0.51}^a^ (0.49–3.46)	1.28 {0.50}^a^ (0.49–3.46)	1.26 {0.54}^a^ (0.72–2.72)	0.669

Normally distributed values were expressed as mean ± standard deviation, otherwise as median (^a^) with interquartile range. Ranges in parentheses. ^†^p-value related to gender. Normally distributed data were tested by the unpaired t-test, otherwise with the Mann–Whitney-U-test. *LV-GLS* left ventricular global longitudinal strain, *LV-GCS* left ventricular global circumferential strain, *LV-GRS* left ventricular global radial strain, *S/D* ratio S-wave to D-wave by quantitative right upper pulmonary vein blood flow measurements, *E/A* ratio E-wave to A-wave by quantitative transmitral blood flow measurements, *E* E-wave by quantitative transmitral blood flow measurements, *A* A-wave by quantitative transmitral blood flow measurements, *é* mean lateral and septal cine CMR velocities, *E/é* ratio E-wave to é, *MAPSE* Mitral annulus plain systolic excursion, *SI* Sphericity index.

**Table 5:** Cardiovascular functional parameter of the subgroup with heart rates between 60 to 75 beats-per-minute.

	All	Women	Men	p†
**All heart rates**				
LV-GLS (%)	− 17.1 ± 1.7 (− 22.3 to − 12.9)	− 17.8 ± 1.6 (− 22.3 to − 14.4)	− 16.1 ± 1.2 (− 18.3 to − 12.9)	< 0.001
LV-GCS (%)	− 19.5 ± 1.9 (− 24.2 to − 15.3)	− 20.2 ± 1.8 (− 24.2 to − 15.6)	− 18.6 ± 1.6 (− 21.8 to − 15.3)	< 0.001
LV-GRS (%)	34.4 {6.9}^a^ (24.0–52.1)	37.1 ± 5.6 (24.0–52.1)	32.2 ± 4.5 (24.2–41.5)	< 0.001
S/D	1.12 {0.59}^a^ (0.58–2.48)	1.11 {0.58}^a^ (0.58–2.48)	1.13 {0.61}^a^ (0.59–2.33)	0.811
E/A	1.84 {0.87}^a^ (0.81–5.14)	1.84 {0.86}^a^ (0.81–5.14)	1.93 ± 0.59 (0.86–3.33)	0.985
E (cm/s)	52.6 ± 9.3 (25.4–76.2)	53.3 ± 9.6 (25.4–76.2)	51.8 ± 8.9 (31.9–70.6)	0.409
A (cm/s)	28.2 {12.8}^a^ (14.8–54.0)	29.2 ± 8.1 (14.8–54.0)	26.6 {13.3}^a^ (18.0–49.8)	0.562
é (cm/s)	13.3 ± 3.7 (5.0–23.0)	12.8 ± 3.6 (5.0–21.9)	14.1 ± 3.9 (6.4–23.0)	0.096
E/é	4.14 {1.41}^a^ (2.13–7.61)	4.18 {1.32}^a^ (2.54–7.61)	3.92 ± 1.04 (2.13–6.11)	0.045
MAPSE (cm)	1.6 ± 0.3 (0.93–2.43)	1.6 ± 0.3 (0.95–2.34)	1.7 ± 0.3 (0.93–2.43)	0.038
SI	1.23 {0.47}^a^ (0.72–3.46)	1.21 {0.44}^a^ (0.72–3.46)	1.26 {0.72}^a^ (0.72–2.45)	0.761

Normally distributed values were expressed as mean ± standard deviation, otherwise as median (^a^) with interquartile range. Ranges in parentheses. ^†^p-value related to gender. Normally distributed data were tested by the unpaired t-test, otherwise with the Mann–Whitney-U-test. *LV-GLS* left ventricular global longitudinal strain, *LV-GCS* left ventricular global circumferential strain, *LV-GRS* left ventricular global radial strain, *S/D* ratio S-wave to D-wave by quantitative right upper pulmonary vein blood flow measurements, *E/A* ratio E-wave to A-wave by quantitative transmitral blood flow measurements, *E* E-wave by quantitative transmitral blood flow measurements, *A* A-wave by quantitative transmitral blood flow measurements, *é* mean lateral and septal cine CMR velocities, *E/é* ratio E-wave to é, *MAPSE* Mitral annulus plain systolic excursion, *SI* Sphericity index.

### Bivariate strain correlation

Correlation analyses of strain with a wide range of cardiac parameters were performed and are listed in the Supplementary Table S1 online. Several parameters correlate moderately but statistically significant with strain. As an example, the E wave correlates with the passive measures of reservoir- (LA-S_res_) and conduit strain (LA-S_con_) but not with the active measure of booster strain (LA-S_boo_). In contrast, the A-wave only correlates moderately with LA-S_boo_ (Supplementary Table S1, Table I).

#### Correlation between age and strain

**Figure 1:**
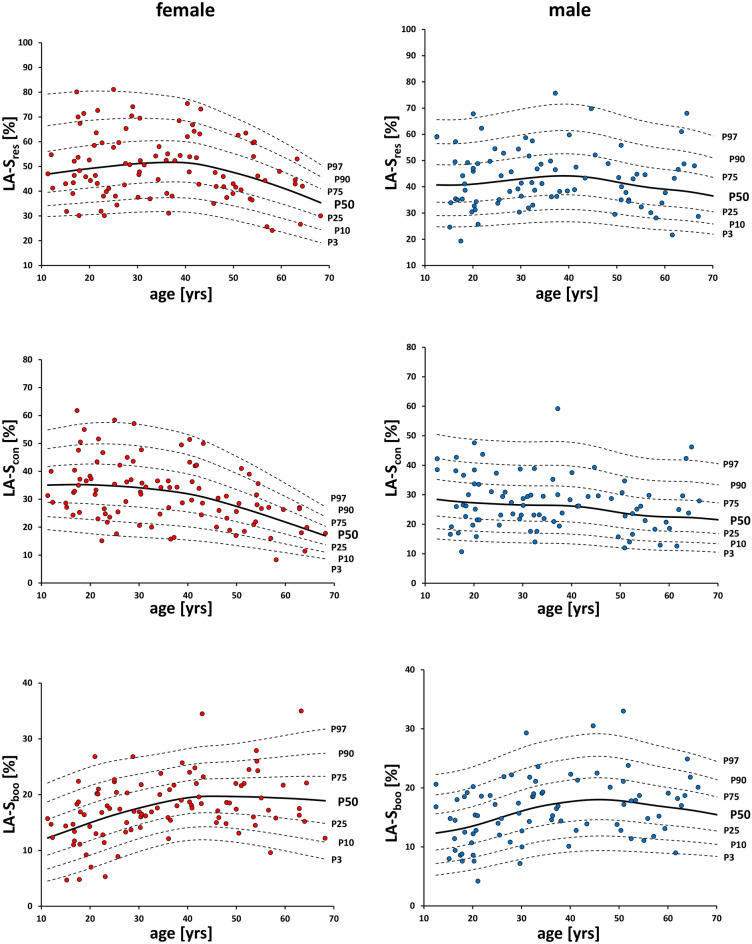
Gender-specific percentile curves of global longitudinal left atrial strain values by CMR feature tracking for reservoir, conduit and booster cardiac phase, respectively. *LA-S*_*res*_ reservoir left atrial strain, *LA-S*_*con*_ conduit left atrial strain, *LA-S*_*boo*_ booster left atrial strain.

The correlations between strain and age are shown as percentiles in Fig. 1 and are summarized in Supplementary Table S1 (Tables III, IV) online. The female conduit strain correlates negatively with age, while the female booster strain correlates positively with age, both at a 0.01 significance level. The male booster strain correlates positively with age at a 0.05 significance level. Although not all strains correlate significantly, the trend dynamics imply a point of inflection at age 40–45 for both sexes.

#### Correlation between age and strain rate

**Figure 2:**
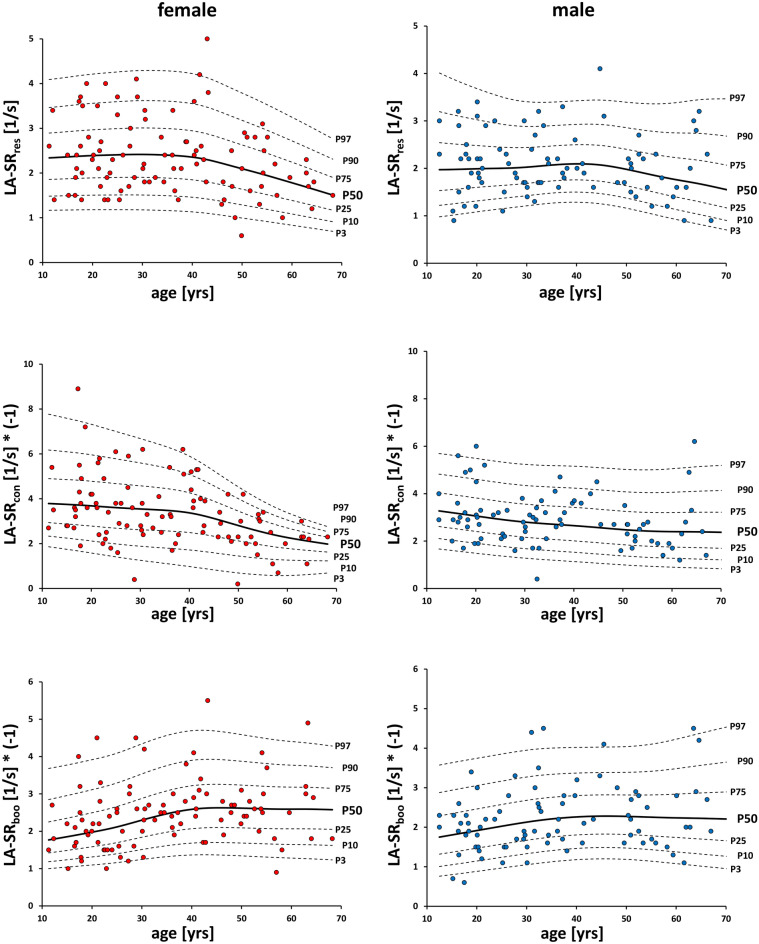
Gender-specific percentile curves of global longitudinal left atrial strain rate values by CMR feature tracking for reservoir, conduit and booster cardiac phase, respectively. *LA-SR*_*res*_ reservoir left atrial strain rate, *LA-SR*_*con*_ conduit left atrial strain rate, *LA-SR*_*boo*_ booster left atrial strain rate.

Similarly to strain percentiles, strain rate percentiles show an inflection point at 40–45 years of age (Fig. 2). Female reservoir and booster strain rates correlate negatively with age, while a positive correlation is observed for female conduit strain rate and age. For males, the conduit strain rate correlates positively with age. The correlations are summarized in Supplementary Table 1 (Tables V, VI) online.

### Multiple linear regression analyses

Multiple linear regression analyses derived from the bivariate correlation analyses are summarized in the Supplementary Tables S2a–d online. Individual cardiac parameters were selected for clustering based on their bivariate value in order to acquire a broad statistical overview of relevant parameters impacting left atrial strain (see also Fig. 4). Due to the demonstrated multifactorial influences of left atrial strain, clusters of selected parameters may better characterize strain function. Multiple linear regression analyses were performed for strain and strain rate of all subjects and subgroups with heart rates between 60 and 75 bpm.

#### All subjects

A sub-selection of multiple linear regression analyses of all subjects is summarized in Table 6. Interestingly, the left atrial reservoir- and conduit strain correlate moderately with the E-wave and corresponding left ventricular strain, among others (e.g. Clusters 2 and 4). In contrast, contractile parameters such as left atrial booster ejection fraction (LA-EF_boo_) and the A wave (e.g. Cluster 8) show moderate (males) to large (females) correlation with left atrial booster strain (LA-S_boo_). A comprehensive collection of the multiple linear regression analyses is given in Supplementary Tables S2a,b online.

**Table 6:** Multilinear regression analysis to study multifactorial influences of left atrial strain. Analysis was done on the total study group. A sub-selection is shown. A comprehensive summary of all testings is provided in the supplement online.

	Validity^a^		Parameter	Sex	R^2^	corr R^2^	ANOVA significance	Sample size^b^	Goodness-of-fit	Function
Reservoir	**2**	LA-S_res_	E, LV-GCS LA-EF_tot_	f	**0.158**	0.131	0.001	**80**	Medium	LA-S_res_ = 0.659 × LA-EF_tot_ + 1.946 × LV-GCS + 0.152 × E + 44.010
	*2*	LA-S_res_	E, LV-GCS LA-EF_tot_	m	**0.205**	0.172	0.001	**60**	Medium	LA-S_res_ = 0.521 × LA-EF_tot_ − 0.891 × LV-GCS + 0.187 × E − 11.379
Conduit	**4**	LA-S_con_	LA-Vol_boo_i_, LA-EF_tot_, LV-GRS E	f	**0.265**	0.233	< 0.001	**48**	Large	LA-S_con_ = − 0.144 × LA-V_boo_i_ + 0.463 × LA-EF_tot_ − 0.613 × LV-GRS + 0.270 × E + 18.627
	**4**	LA-S_con_	LA-Vol_boo_i_, LA-EF_tot_, LV-GRS E	m	**0.225**	0.181	0.001	**59**	Medium	LA-S_con_ = − 0.124 × LA-V_boo_i_ + 0.418 × LA-EF_tot_ + 0.064 × LV-GRS + 0.175 × E − 2.450
Booster	*8*	LA-S_boo_	LA-EF_boo_, LV-ESV_i_, age A	f	**0.306**	0.275	< 0.001	**41**	Large	LA-S_boo_ = 0.315 × LA-EF_boo_ + 0.307 × LV-ESV_i_ + 0.100 × age + 0.008 × A − 4.055
	**8**	LA-S_boo_	LA-EF_boo_, LV-ESV_i_, age A	m	**0.200**	0.156	0.002	**67**	Medium	LA-S_boo_ = 0.106 × LA-EF_boo_ − 0.292 × LV-ESV_i_ + 0.025 × age + 0.039 × A + 18.547

In order to be able to carry out a multilinear regression analysis, it is mandatory to check the prerequisites in advance. These are (a) linear relationships between the variables, (b) no outliers, (c) independence of the residuals, (d) no multicollinearity, (e) homoscedasticity and (f) normal distribution.

^a^Bold = prerequisites are met; italics = valid, but with fewer concerns due to the prerequisites (e.g. an outlier had to be removed or the independence of the residuals had not reached the optimal value of ~ 2 before the analysis).

Number of females = 97; number of males = 86.

Interpretation of |R^2^| according to Cohen. [Cohen, J. (1988). Statistical power analysis for the behavioral sciences (2nd ed.). Hillsdale, N.J.: L. Erlbaum Associates. Page 412 ff.].

Small effect size:|R^2^|= 0.02; medium effect size |R^2^|= 0.13; large effect size |R^2^|= 0.26.

^b^Sample size calculation see reference [Calculation according to Hemmerich, W. (2019). StatistikGuru: Poweranalyse und Stichprobenberechnung für Regression. Retrieved from https://statistikguru.de/rechner/poweranalyse-regression.html].

*LA-S*_*res*_ reservoir left atrial strain, *LA-S*_*con*_ conduit left atrial strain, *LA-S*_*boo*_ booster left atrial strain, *LA-Vol*_*boo_i*_ indexed booster left atrial volume, *LA-EF*_*total*_ total left atrial ejection fraction, *LA-EF*_*boo*_ booster left atrial ejection fraction, *LV-GCS* global left ventricular circumferential strain, *LV-GRS* global left ventricular radial strain, *E* E-wave by quantitative transmitral blood flow measurements, *A* A-wave by quantitative transmitral blood flow measurements, *LV-ESV*_*i*_ indexed left ventricular end systolic volume.

#### Subgroup with HR 60–75 bpm

Identical clustering was used for the subgroup with selected heart rates between 60 and 75 bpm. A sub-selection of multiple linear regression analyses is summarized in Table 7. In contrast to all subjects, the subgroup with a heart rate of 60–75 bpm generally showed higher multiple correlation coefficients R^2^ for the overall model between the selected cardiac parameters and left atrial strain or left atrial strain rate, indicating improved goodness-of-fit according to Cohen^23^. For example, the R^2^ value for cluster 2 was 0.158 for females and 0.205 for males when all subjects were included in the analysis, whereas a higher R^2^ value of 0.278 for females and a higher R^2^ value of 0.320 for males was found for the subgroup. A comprehensive summary of the multiple linear regression analyses performed in the subgroups is given in Supplementary Table S2c,d.

**Table 7:** Multilinear regression analysis to study multifactorial influences of left atrial strain. Analysis was done on subjects with heart rates between 60 and 75 bpm. A sub-selection is shown in this table. A comprehensive summary of all analyses is provided in the supplement online.

	Validity^a^		Parameter	Sex	R^2^	corr R^2^	ANOVA significance	Sample size^b^	Goodness-of-fit	Function
Reservoir	**2**	LA-S_res_	E, LV-GCS LA-EF_tot_	f	**0.278**	0.238	< 0.001	41	Large	LA-S_res_ = 0.825 × LA-EF_tot_ + 2.633 × LV-GCS + 0.250 × E + 44.270
	**2**	LA-S_res_	E, LV-GCS LA-EF_tot_	m	**0.320**	0.267	0.002	35	Large	LA-S_res_ = 0.897 × LA-EF_tot_ − 1.114 × LV-GCS + 0.212 × E − 36.764
Conduit	**4**	LA-S_con_	LA-Vol_boo_i_, LA-EF_tot_, LV-GRS E	f	**0.437**	0.395	< 0.001	26	Large	LA-S_con_ = 0.185 × LA-V_boo_i_ + 0.931 × LA-EF_tot_ − 1.056 × LV-GRS + 0.351 × E − 5.403
	**4**	LA-S_con_	LA-Vol_boo_i_, LA-EF_tot_, LV-GRS E	m	**0.405**	0.341	0.001	28	Large	LA-S_con_ = − 0.004 × LA-V_boo_i_ + 0.458 × LA-EF_tot_ + 0.030 × LV-GRS + 0.437 × E − 21.650
Booster	**8**	LA-S_boo_	LA-EF_boo_, LV-ESV_i_, age A	f	**0.418**	0.374	< 0.001	27	Large	LA-S_boo_ = 0.430 × LA-EF_boo_ + 0.286 × LV-ESV_i_ + 0.116 × age + 0.138 × A − 11.988
	*8*	LA-S_boo_	LA-EF_boo_, LV-ESV_i_, age A	m	**0.217**	0.139	0.040	***61***	Medium	LA-S_boo_ = 0.209 × LA-EF_boo_ − 0.259 × LV-ESV_i_ − 0.008 × age + 0.120 × A + 13.311

In order to be able to carry out a multilinear regression analysis, it is mandatory to check the prerequisites in advance. These are (a) linear relationships between the variables, (b) no outliers, (c) independence of the residuals, (d) no multicollinearity, (e) homoscedasticity and (f) normal distribution.

^a^Bold = prerequisites are met; italics = valid, but with fewer concerns due to the prerequisites (e.g. an outlier had to be removed or the independence of the residuals had not reached the optimal value of ~ 2 before the analysis); bolditalics = not valid due to violation of prerequisites.

Number of females = 60; number of males = 46.

Interpretation of |R^2^| according to Cohen. [Cohen, J. (1988). Statistical power analysis for the behavioral sciences (2nd ed.). Hillsdale, N.J.: L. Erlbaum Associates. Page 412 ff].

Small effect size: |R^2^|= 0.02; medium effect size |R^2^|= 0.13; large effect size |R^2^|= 0.26. ^b^Sample size calculation see reference [Calculation according to Hemmerich, W. (2019). StatistikGuru: Poweranalyse und Stichprobenberechnung für Regression. Retrieved from https://statistikguru.de/rechner/poweranalyse-regression.html].

*LA-S*_*res*_ reservoir left atrial strain, *LA-S*_*con*_ conduit left atrial strain, *LA-S*_*boo*_ booster left atrial strain, *LA-Vol*_*boo_i*_ indexed booster left atrial volume, *LA-EF*_*total*_ total left atrial ejection fraction, *LA-EF*_*boo*_ booster left atrial ejection fraction, *LV-GCS* global left ventricular circumferential strain, *LV-GRS* global left ventricular radial strain, *E* E-wave by quantitative transmitral blood flow measurements, *A* A-wave by quantitative transmitral blood flow measurements, *LV-ESV*_*i*_ indexed left ventricular end systolic volume.

### Intra- and interobserver variability

Intra- and interobserver variability was tested on 20 randomized subjects. All intra- and interobserver variability resulted in excellent intra-class-correlation coefficients (ICC > 0.9) and low coefficient of variation (CoV) for intra- and interobserver variability of < 10%. The highest intra- and inter-observer resemblance were for reservoir strain (ICC 0.967 and 0.965), whereas the lowest intra- and inter-observer variations was for booster strain (ICC 0.941 and 0.936). The lowest bias and scatter by Bland–Altman statistics was found for the intra-observer reservoir strain, with a mean difference (95% confidence interval) of 2.0% (− 10.7 to 14.6%) whereas the greatest bias and scatter was found for the inter-observer conduit strain, with a mean difference of 6.3% (− 30.2 to 42.8%).

## Discussion

Several recent studies have defined reference values for left atrial strain. However, fundamental differences in age- and sex-dependent strain emphasize the need to reevaluate the clinical factors influencing strain and identify new diagnostic concepts.

The influence of heart rate on LA strain and strain rate was initially demonstrated in this study by performing the bivariate product moment correlation (see Supplement S1). Based on this observation, novel aspects of our study include the analysis of heart rate on the left atrial strain and the use of multilinear regression analysis for use in multi-parametric diagnostic. Moreover, this CMR-study yields normal left atrial strain and strain rate values based on a large cohort with a broad age range between 10 and 70 years. The main findings of this study are: (I) subjects with a heart rate of 60–75 beats per minute do not show gender differences in strain and strain rate, (II) novel parameter clusters allow improved multiparametric characterization of strain and strain rate, (III) multiparametric analyses achieved a higher correlation value in the heart rate subgroup of 60–75 beats per minute, and (IV) percentile plotting imply changes in cardiac remodeling at age 40–45 years.

### Influence of heart rate on left atrial strain

As shown in Supplement S1, Table I, left atrial reservoir strain and left atrial conduit strain increase with higher heart rate in the total cohort by bivariate linear regression analysis. This is in line with a recent MRI study by Weise Valdés et al. in which an increase in global longitudinal, circumferential and radial strain with increasing heart rate was also observed, but in the left ventricle of healthy subjects^15^. Consistent with our observations, they found significantly elevated left ventricular strain in women in contrast to men. Upon conducting a mediator analysis between the impact of sex or indexed muscle mass on the strain, they identified indexed muscle mass as a partial mediator variable in contrast to gender. These observations complement our findings, implying the impact of gender on strain may be inferior to factors such as heart rate or indexed muscle mass.

Within our study, limiting the participant selection to a heart rate spectrum of 60–75 bpm, no gender differences were found for all functional phases of strain and strain rate, although neither strain nor strain rate differed statistically significantly between the two cohorts. Consistent findings were demonstrated by Truong et al. Although their study did not systematically filter participants by heart rate, this observation can be explained given the narrow range of their participants' mean heart rate of 63 ± 10 bpm^13^. As previously demonstrated^15^, cardiac strain could vary depending upon patient heart rate, important for diagnostic implications. An increased heart rate at rest is generally discussed as a risk factor for chronic heart failure^16^, which may explain strain deviation from a normal heart rate collective. Although not discussed in this reference, heart rate-controlled strain may be a very useful feature to confirm this assumption. Additionally, the influence of gender may be secondary when considering heart rate-dependent changes in strain and strain rate.

### Age- and gender related left atrial strain

Although large cohort studies of left atrial strain have been previously conducted, study results^9,10,13,14^ have remained incongruent for age and gender associated functional strain phases. Consistent with Truong et al.^13^, our CMR study found no difference in reservoir strain with age for both genders. However, Qu et al. found a decreasing reservoir strain with age for both genders^14^. Both Qu et al., and Truong et al. observed decreasing conduit function over age, which we observed only for females. It is important to note that these inconsistencies can be attributed to differences in modalities used^17^ and/or software quantification techniques^1^. Furthermore, it is possible that with increasing age, alterations in cardiac fibrotic remodeling may occur^18^ impairing left ventricular relaxing^19^, in turn reducing left atrial conduit strain. In our study, an increase in booster strain with age was observed in both genders, which is consistent with Truong et al. and Liao et al.^9,13^. In contrast, other authors did not observe any influence of age on booster strain^10,14,20^. Although we found an overall increase in booster strain, the percentiles for men and women show a plateau phase followed by a decline after the age of 45, which is more pronounced in men. A likely explanation for this development in the second half of life may be due to atrial dilation, which may expand the left atrial stroke volume beyond the Frank-Starling relationship^21,22^. The discussed incongruities demonstrate the need for a novel diagnostic approach, as strain appears to be subject to fluctuation independent of age and gender. Integrating strain into parametric clusters may better characterize the complexity of cardiac hemodynamics and deformation.

### Multiple linear regression analyses

To our knowledge, we are the first to perform a comprehensive assessment of the determinants of left atrial strain using multiple linear regression. The wealth of data attained with a single cardiovascular magnetic resonance examination requires a multiparametric approach for improved diagnostic differentiation. Concerning Cohens interpretation of effect size^23^, we found multiple correlations with “medium” to “large” effect sizes. The correlation may be explained using the principles of cardiac physiology. Per example, the passive reservoir phase can be considered a volumetric summation of both conduit and booster phase^24^, correlating with total left atrial ejection fraction. The reservoir phase is characterized by atrial filling via blood inflow from the pulmonary veins and is thus representative of chamber compliance. As the majority of left atrial volume is ejected in healthy subjects, its correlation with reservoir deformation is implied. Furthermore, greater atrial volumetric filling and distensibility are expected to be mirrored within the left ventricular circumferential strain, as the ventricular filling will alter ventricular circumferential deformation.

With the exception of males in booster phase, all correlations of the heart rate controlled subgroup exhibited a “large” effect size. This is mainly due to the improved prerequisites for performing statistical multiple linear regression analyses (e.g. fewer outliers that had to be excluded), which increases the reliability of the analysis.

Extracardiac factors, such as aging, are additionally included to account for age-related cardiac changes. Booster function in particular has been described to increase, in order to compensate for the reduction in reservoir function associated with age^13^, which is consistent with our multiparametric results.

A recent CMR-study used multiparametric analyses of the left ventricle to accurately differentiate diagnostically between healthy subjects, athletes, hypertensive heart disease, hypertrophic cardiomyopathy and cardiac amyloidosis^25^. We present reference clusters and their correlative value for healthy left atrial strain with subsequent heart rate-adjusted reference values. These bear promising clinical implications and remain to be assessed under disease state.

### Future perspectives

Clinical utility of MRI-attained LA strain has experienced extensive growth in its diagnostic value. For example, the challenge of distinguishing between hypertrophic cardiomyopathy from cardiac amyloidosis^6,26,27^ or even the differentiation between subtypes of amyloidosis^24^ were shown to be facilitated through left atrial strain quantification. The majority of previous studies were performed using echocardiography, whereby MRI data still remains limited and partially inconsistent, particularly regarding specific parameter dependencies (e.g. age^9,13,14^) between functional phases reservoir, conduit and booster. Differentiating healthy from pathological state at early stage of disease, remains the primary clinical application of normal strain values. Apart from providing normal left atrial strain parameters and exploring strain dependencies between age, sex and heart rate, this study seeks to provide a novel perspective by discovering novel dependencies, which have been conceptualized in a multiparametric design. As indicated in Tables 6 and 7, adaption of these multiparametric clusters in future studies may require a smaller sample size in heart rate controlled subjects, raising cost-effectiveness and statistical validation.

### Intra- and inter-observer variation

High resemblance in intra- and inter-observer assessment reflect the reliability for left atrial strain quantification using CMR, particularly for reservoir strain. Conduit strain has been shown to be the most difficult to determine, possibly due to variations in downward sloping. These resemblances however, can be expected to improve further, as CMR and its associated software quantifications and automatic contouring continue to progress. Although comparable intra- and inter-observer variations have been presented by a recent speckle tracking echocardiographic study (STE), the far field location of the left atrium and its thin wall remain a challenge to the STE modality^10^. A further CMR study observed superior reproducibility of CMR in contrast to echocardiographic examination^13^.

### Limitations

This study is a single-center study. We present novel multiparametric dependencies developed on the basis of our own statistical analyses. Their clinical value requires further validation in large cohort studies. Furthermore, no intermodal comparison, for example comparison between echocardiographic and CMR attained values, was carried out. Furthermore, the maximum heart rate (HR) of 111 beats per minute (bpm) of a child aged 15 remained included, considering it was nervous throughout its first CMR examination, even if this value lies slightly outside the 99th percentile age corresponding heart rate^28^. Lastly, the angulation of the 2- and 4-chamber views represent a further limitation, in which variations in the maximum longitudinal axis at end-systole occur due to the convulsive movement of the heart, even after careful planning at end-diastole.

## Conclusion

This CMR-study yields normal left atrial strain and strain rate values for a wide age range of healthy subjects and is the first to demonstrate the impact of the heart rate on left atrial strain. Heart rate control appears to reduce gender differences in strain. In addition, this is associated with improved prerequisites for performing statistical multiple linear regression analyses, which increases the reliability of the analysis. Ultimately, our multiparametric regression analyses offer an innovational diagnostic concept to complement multi-parametric CMR analyses for characterization of left atrial strain.

## Methods

For the purpose of this study 208 subjects were initially recruited. The local ethics committee approved the study conditions (Ethikkommission der Medizinischen Fakultät der Ruhr-Universität Bochum, Sitz Bad Oeynhausen, registration number: 2017-238). All examinations were done in accordance with the 1964 declaration of Helsinki. Prior to inclusion in the study, a written informed consent was obtained from all participants or their legal guardians in the case of participants < 18 years of age.

In order to limit the study to healthy participants only, a health assessment questionnaire was carried out beforehand. Exclusion criteria entailed clinical history of cardiovascular disease and surgery, medication for cardiovascular or metabolic disorders, associated risk factors and contraindications for CMR. After all inclusion criteria were met, the CMR assessment was performed. If CMR imaging demonstrated myocardial abnormalities, aortic ectasia, pulmonary trunk dilation, valvular heart disease, ischemic heart disease, signs of cardiomyopathy the individuals would be excluded. Based on these criteria 22 volunteers were excluded. Furthermore, three subjects were rejected from the strain analysis because of insufficient image quality. The final study group thus comprised 183 healthy individuals that participated in a CMR study.

### Cardiovascular magnetic resonance imaging

CMR Imaging was performed using a multi-transmit 3.0 Tesla magnetic resonance imaging system (Achieva, Philips Healthcare, Best, The Netherlands; Release 5.3.1 and 5.6.1, respectively) with dStream technology. All volunteers were examined in supine position. In order to enable cardiac-triggering acquisitions, a vector electrocardiogram was applied. The maximum gradient performance was 40 mT/m, slew rate = 200 mT/m/ms and signal reception was achieved using a cardiac phased-array coil. The standard examination protocol included 2-chamber and 4-chamber long-axis views, a 2D stack of axially acquired slices covering the whole heart as well as a short-axis 2D stack covering the entire left and right ventricles (12–16 slices, no gap) utilizing cine steady-state free-precession acquisitions (TR/TE/flip angle = 2.7 ms/1.35 ms/42°) to assess cardiac function, morphology and strain. Twenty-eight or 45 cardiac phases were collected per cardiac cycle. Assuming an averaged heart rate of 67 bpm, temporal resolution was 32 or 20 ms per cardiac phase, respectively. Spatial resolution was 1.5 × 1.5 × 8 mm^3^.

A conventional flow-sensitive, retrospectively triggered gradient-echo pulse sequence (TR/TE/flip angle = 10 ms/4.2 ms/30°) was applied to quantify transmitral and right upper pulmonary venous blood flow. Through-plane blood flow measurements were performed with velocity-encoded values of 70–100 cm/s. A SENSE-reduction factor of 2 was applied. In order to resolve the fine structure of the pulmonary venous flow profiles in particular, a temporal resolution of 10 ms was chosen. Therefore, the number of phases was always adjusted to the subject’s individual heart rate. Quantitative blood flow evaluation was performed offline on a computer workstation using the homemade “HDZ MR-Tools” software package (HDZ, Bad Oeynhausen, Germany).

### Strain analysis

**Figure 3:**
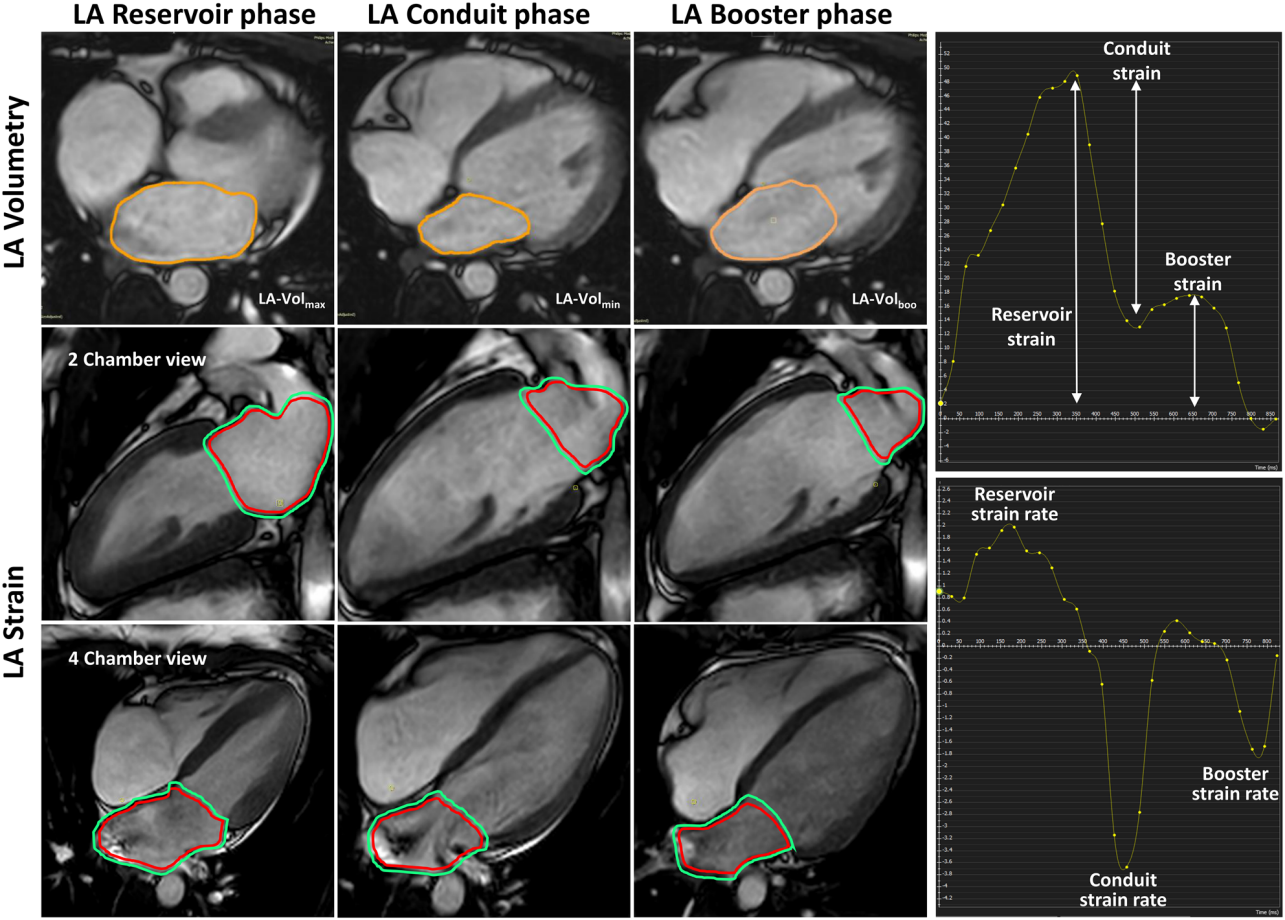
Measurement of left atrial longitudinal strain and strain rate in a 24-year-old healthy female. Upper row: Determination of left atrial chamber volumes in the reservoir, conduit and booster cardiac phase, respectively. The entire axial stack of slices was used for the volume calculations. Accordingly, endocardial (red) and epicardial (green) left atrial contours are shown in the two-chamber view (middle row) and in the four-chamber view (lower row) for strain estimation in the corresponding cardiac phases. Upper right: Left atrial strain curve. Lower right: Left atrial strain rate curve.

Longitudinal strain was expressed in negative values. Thus, when describing an "increase" or a "higher value" an increase in negativity is meant. Strain analysis was conducted using the CVI42^®^ software package (Circle Cardiovascular Imaging Inc., Calgary, Canada, Release 5.12.1). Endocardial and epicardial contours of the left atrium in 2-chamber and 4-chamber long-axis slices were delineated manually in the end-diastolic heart frame (Fig. 3) and subsequently followed by automatic registration of the applied software. The linings excluded the ostiums of the pulmonary veins as well as the left atrial appendage. The total global longitudinal strain (GLS) as well as individual global longitudinal strain for 4-chamber and 2-chamber were quantified.

**Figure 4:**
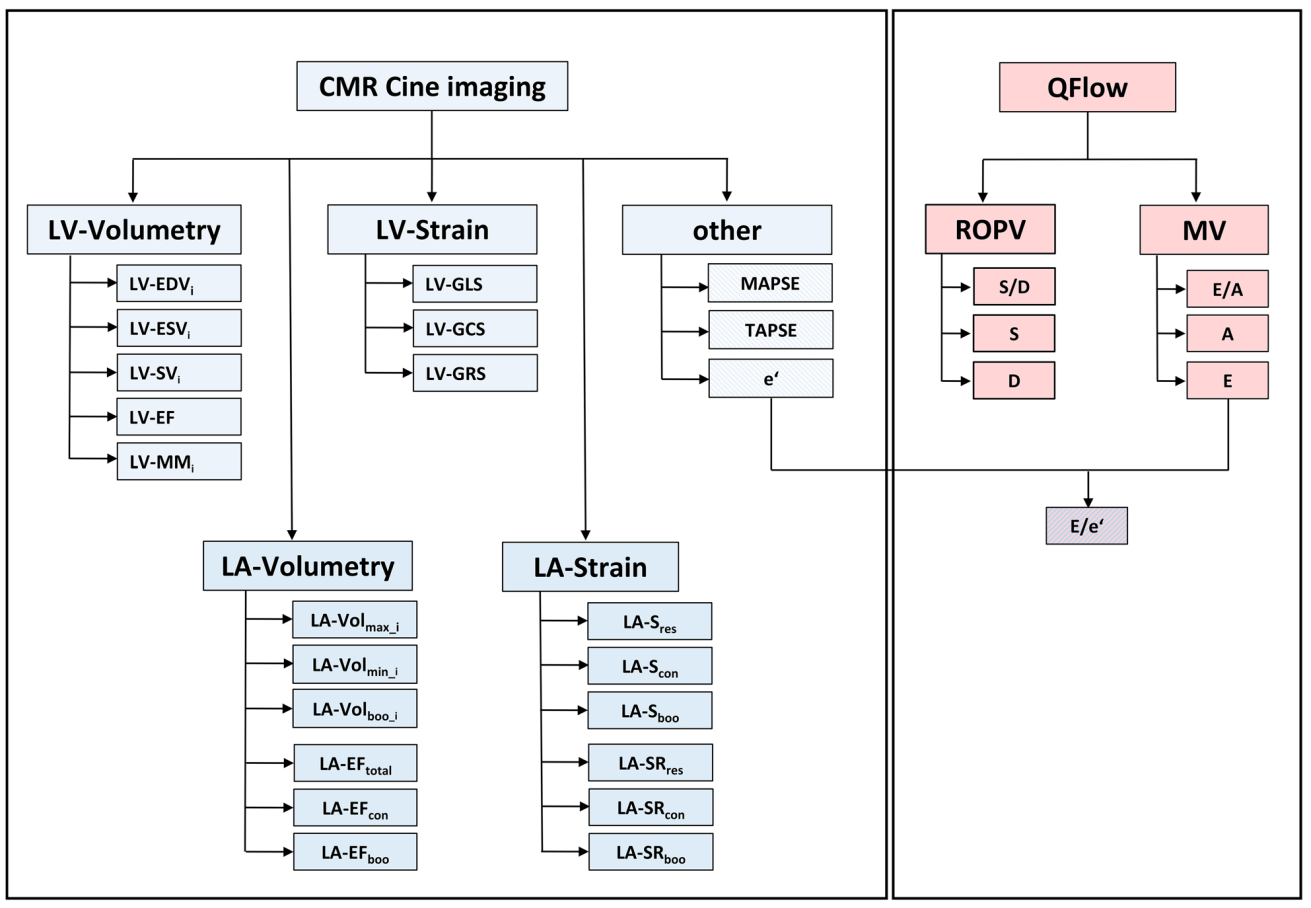
Overview of measures for investigation of the correlations between left atrial strain/strain rate and volumetric, hemodynamic and functional parameters. Left atrial and left ventricular volumetric data as well as strain/strain rate values were calculated using cine cardiovascular magnetic resonance (CMR) imaging. Systolic and diastolic functional parameters were obtained applying quantitative blood flow measurements (QFlow). *LA-S*_*res*_ reservoir left atrial strain, *LA-S*_*con*_ conduit left atrial strain, *LA-S*_*boo*_ booster left atrial strain, *LA-SR*_*res*_ reservoir left atrial strain rate, *LA-SR*_*con*_ conduit left atrial strain rate, *LA-SR*_*boo*_ booster left atrial strain rate, *LA-Vol*_*max_i*_ indexed maximum left atrial volume, *LA-Vol*_*min_i*_ indexed minimum left atrial volume, *LA-Vol*_*boo_i*_ indexed booster left atrial volume, *LA-EF*_*total*_ total left atrial ejection fraction, *LA-EF*_*con*_ passive left atrial ejection fraction, *LA-EF*_*boo*_ booster left atrial ejection fraction, *LV-EDV*_*i*_ indexed left ventricular end diastolic volume, *LV-ESV*_*i*_ indexed left ventricular end systolic volume, *LV-SV*_*i*_ indexed left ventricular stroke volume, *LV-EF* left ventricular ejection fraction, *MM*_*i*_ BSA-indexed muscle-mass, *LV-GLS* global left ventricular longitudinal strain, *LV-GCS* global left ventricular circumferential strain, *LV-GRS* global left ventricular radial strain, *MAPSE* mitral annulus plain systolic excursion, *TAPSE* trikuspidal annulus plain systolic excursion, *S/D* ratio S-wave to D-wave by quantitative right upper pulmonary vein blood flow measurements, *E/A* ratio E-wave to A-wave by quantitative transmitral blood flow measurements, *E* E-wave by quantitative transmitral blood flow measurements, *A* A-wave by quantitative transmitral blood flow measurements, *é* mean lateral and septal cine CMR velocities, *E/é* ratio E-wave to é.

A comprehensive set of different measures was analyzed to investigate the correlations between left atrial strain/strain rate and volumetric, hemodynamic and functional parameters (Fig. 4 and Supplementary Table S1). These comprise left atrial end-diastolic volume (LA-Vol_min_i_), booster volume (LA-Vol_boo_i_), end-systolic volume (LA-Vol_max_i_) as well as the corresponding derived ejection fractions (LA-EF_tot_, LA-EF_con_ and LA-EF_boo_) which were obtained by defining the endocardial contours in the axially acquired cine stack and applying the Simpson approach. Global left ventricular longitudinal strain (LV-GLS), global left ventricular circumferential strain (LV-GCS) and global left ventricular radial strain (LV-GRS) as well as the BSA-indexed end-diastolic left ventricular volume (LV-EDV_i_), BSA-indexed end-systolic left ventricular volume (LV-ESV_i_), BSA-indexed stroke volume (LV-SV_i_) and BSA-indexed left ventricular muscle mass (MM_i_) were estimated based on the cine short axis steady-state free-precession acquisitions. The passive early diastolic filling of the left ventricle (E-wave), the active late diastolic filling of the left ventricle (A-wave) and the diastolic transmitral flow velocity ratio E/A were obtained by quantitative transmitral blood flow measurements. The cine 4-chamber view was used to assess the early diastolic mitral annular tissue velocity é allowing the calculation of the ratio E/é. The pulmonary venous flow ratio S/D defined as the forward flow during ventricular systole (S) to the early diastole (D) was recorded to assess diastolic function.

### Statistical analysis

Statistical analysis was carried out utilizing IBM SPSS Statistics (version 26.0.0.0, IBM Deutschland GmbH). Normal distribution was tested using Shapiro–Wilk test. Continuous variables were presented as mean ± standard deviation (SD) when normally distributed, otherwise as median with interquartile range. Differences in continuous variables between baseline parameters, cardiac function and left atrial strain of men and women were evaluated using an unpaired Student’s t-test for normal distribution and the Mann–Whitney-U test for non-normal distribution. Correlations between variables were tested by bivariate linear regression analysis. The decision to use the Pearson product-moment correlation or the Spearman’s Rho correlation was made after reviewing the prerequisites for a linear regression analysis such as linearity of the data, checking for outliers via box plots and normal distribution. The relationship between two or more independent variables and a dependent variable with previously established significant correlations was determined by multilinear regression analysis. The effect size |R^2^| was interpreted according to Cohen^23^ with the following grading: small effect size: |R^2^|= 0.02; medium effect size: |R^2^|= 0.13 and large effect size: |R^2^|= 0.26. In this study, only correlations were shown after fulfilling the prerequisites for performing multilinear regression analysis including linearity, checking for outliers, independence of residuals, multicollinearity, homoscedasticity and normal distribution according to Hemmerich^29^ (version 1.96). Inter-observer and intra-observer variability was tested by Bland-Altmann analysis, intra-class-correlation coefficients (ICC, two-way mixed model, absolute agreement^30^ and coefficients of variation (CoV). In general, a p value < 0.05 was considered statistically significant. The LMS method^31^ was applied for generating age-dependent and sex-specific percentile curves of left atrial strain values, using the LMS software^32^ (version 2.54) for fitting.

## Supplementary Information

Supplementary Information.
