# Supplementary material for: Multi-parametric analyses to investigate dependencies of normal left atrial strain by cardiovascular magnetic resonance feature tracking
Source: Sci Rep. 2022 Jul 18;12:12233. doi: 10.1038/s41598-022-16143-0 (PMC9293891; doi:10.1038/s41598-022-16143-0)
Supplement: Supplementary file 1 — Supplementary Information. [file 41598_2022_16143_MOESM1_ESM.pdf]

Multi-parametric analyses to investigate dependencies of normal left atrial strain by cardiovascular magnetic resonance feature tracking

Jan Eckstein, Hermann Körperich, Lech Paluszkiewicz, Wolfgang Burchert, Misagh Piran

Supplement S1

Correlations of left atrial strain

I. Correlations between left atrial *strain* measures and volumetric, hemodynamic and functional parameters – total cohort

Interpretation of |r| or |ρ| according to Cohen

Cohen, J. (1988). Statistical power analysis for the behavioral sciences (2nd ed.). Hillsdale, N.J.: L. Erlbaum Associates.

small effect size	|r| or |ρ| = .10
medium effect size	|r| or |ρ| = .30
large effect size	|r| or |ρ| = .50

Calculation according to Hemmerich, W. (2019). StatistikGuru: Poweranalyse und Stichprobenberechnung für Regression. Retrieved from https://statistikguru.de/rechner/poweranalyse-regression.html

Total (N = 183)	**LA-S_{res}**	**LA-S_{con}**	**LA-S_{boo}**
**HR**	0.150§	0.214*	-0.039
**LA-EF_{tot}**	0.273*	0.310*	0.085
**LV-EF**	0.024	-0.045	0.162§
**LV-GLS**	-0.068	-0.015	-0.122
**LV-GCS**	-0.084	-0.006	-0.148§
**LV-GRS**	0.080	0.000	0.154§
**S/D**	-0.046	-0.204*	0.301*
**E/A**	0.061	0.220*	-0.267*
**E**	0.245*	0.340*	-0.068
**A**	0.052	-0.068	0.241*
**E/e‘**	0.103	0.080	0.048

**e'**	0.023	0.096	-0.080
**MAPSE**	0.105	0.148§	-0.025
**SI**	-0.084	-0.014	-0.120
**Age**	-0.096	-0.294*	0.313*
**MM_i**	-0.314*	-0.267*	-0.216*

*Statistically significant ($p < 0.01$, two-sided).
§Statistically significant ($p < 0.05$, two-sided).
$LA\text{-}S_{res}$, reservoir left atrial strain; $LA\text{-}S_{con}$, conduit left atrial strain; $LA\text{-}S_{boo}$, booster left atrial strain; HR, heart rate; $LA\text{-}EF_{total}$, total left atrial ejection fraction; LV-EF, left ventricular ejection fraction; LV-GLS, global left ventricular longitudinal strain; LV-GCS, global left ventricular circumferential strain; LV-GRS, global left ventricular radial strain; S/D, ratio S-wave to D-wave by quantitative right upper pulmonary vein blood flow measurements; E/A, ratio E-wave to A-wave by quantitative transmitral blood flow measurements; E, E-wave by quantitative transmitral blood flow measurements; A, A-wave by quantitative transmitral blood flow measurements; é, mean lateral and septal cine CMR velocities; E/é, ratio E-wave to é; MAPSE, Mitral annulus plain systolic excursion; SI, Sphericity index; MM_i, BSA-indexed muscle-mass.

II. Correlations between left atrial *strain rates* and volumetric, hemodynamic and functional parameters – total cohort

Total (N = 183)	$LA\text{-}SR_{res}$	$LA\text{-}SR_{con}$	$LA\text{-}SR_{boo}$
**HR**	0.251*	-0.241*	-0.378*
**$LA\text{-}EF_{tot}$**	0.261*	-0.268*	-0.152§
**LV-EF**	0.007	0.020	-0.133
**LV-GLS**	-0.029	0.025	0.194*
**LV-GCS**	-0.038	0.053	0.267*
**LV-GRS**	0.040	-0.050	-0.274*
**S/D**	-0.118	0.215*	-0.266*
**E/A**	0.098	-0.171§	0.283*
**E**	0.261*	-0.355*	-0.019
**A**	0.017	-0.001	-0.329*
**E/e'**	0.152§	-0.098	-0.087
**e'**	-0.018	-0.084	0.064
**MAPSE**	0.092	-0.035	0.088
**SI**	0.021	0.048	0.026
**Age**	-0.164§	0.308*	-0.231*
**MM_i**	-0.233*	0.283*	-0.291*

*Statistically significant ($p < 0.01$, two-sided).
§Statistically significant ($p < 0.05$, two-sided).
$LA\text{-}SR_{res}$, reservoir left atrial strain rate; $LA\text{-}SR_{con}$, conduit left atrial strain rate; $LA\text{-}SR_{boo}$, booster left atrial strain rate; HR, heart rate; $LA\text{-}EF_{total}$, total left atrial ejection fraction; LV-EF, left ventricular ejection fraction; LV-GLS, global left ventricular longitudinal strain; LV-GCS, global left ventricular circumferential strain; LV-GRS, global left ventricular radial strain; S/D, ratio S-wave to D-wave by quantitative right upper pulmonary vein blood flow measurements; E/A, ratio E-wave to A-wave by quantitative transmitral blood flow measurements; E, E-wave by quantitative transmitral blood flow measurements; A, A-wave by quantitative transmitral blood flow measurements; é, mean lateral and septal cine CMR velocities; E/é, ratio E-wave to é; MAPSE, Mitral annulus plain systolic excursion; SI, Sphericity index; MM_i, BSA-indexed muscle-mass.

III. Correlations between left atrial *strain* measures and volumetric, hemodynamic and functional parameters – male subjects, total cohort

Male (N = 86)	$LA\text{-}S_{res}$	$LA\text{-}S_{con}$	$LA\text{-}S_{boo}$
**HR**	0.237§	0.296*	-0.028
**$LA\text{-}Vol_{max\_i}$**	-0.261§	-0.289*	-0.017
**$LA\text{-}Vol_{min\_i}$**	-0.325*	-0.364*	-0.022
**$LA\text{-}Vol_{boo\_i}$**	-0.288*	-0.350*	0.053
**$LA\text{-}EF_{tot}$**	0.332*	0.351*	0.094
**$LA\text{-}EF_{con}$**	0.193	0.306*	-0.164
**$LA\text{-}EF_{boo}$**	0.211	0.105	0.288*
**LV-EF**	0.173	0.083	0.278*
**LV-GLS**	-0.115	-0.057	-0.172
**LV-GCS**	-0.222§	-0.200	-0.122
**LV-GRS**	0.221§	0.199	0.123
**S/D**	-0.084	-0.227§	0.283*
**E/A**	0.050	0.142	-0.193
**E**	0.235§	0.293*	-0.034
**A**	0.088	0.023	0.209
**E/e'**	0.039	0.035	0.037
**e'**	0.094	0.139	-0.048
**MAPSE**	0.084	0.153	-0.075
**SI**	-0.077	-0.013	-0.184
**BMI**	-0.064	-0.087	-0.014
**$LV\text{-}EDV_i$**	-0.106	-0.008	-0.173
**$LV\text{-}ESV_i$**	-0.184	-0.052	-0.326*
**$LV\text{-}SV_i$**	0.018	0.050	0.007
**Age**	-0.049	-0.193	0.271§
**MM_i**	-0.210	-0.134	-0.147

*Statistically significant ($p < 0.01$, two-sided).

§Statistically significant ($p < 0.05$, two-sided).

$LA\text{-}S_{res}$, reservoir left atrial strain; $LA\text{-}S_{con}$, conduit left atrial strain; $LA\text{-}S_{boo}$, booster left atrial strain; HR, heart rate; $LA\text{-}Vol_{max\_i}$, indexed maximum left atrial volume; $LA\text{-}Vol_{min\_i}$, indexed minimum left atrial volume; $LA\text{-}Vol_{boo\_i}$, indexed booster left atrial volume; $LA\text{-}EF_{total}$, total left atrial ejection fraction; $LA\text{-}EF_{con}$, passive left atrial ejection fraction; $LA\text{-}EF_{boo}$, booster left atrial ejection fraction; LV-EF, left ventricular ejection fraction; LV-GLS, global left ventricular longitudinal strain; LV-GCS, global left ventricular circumferential strain; LV-GRS, global left ventricular radial strain; S/D, ratio S-wave to D-wave by quantitative right upper pulmonary vein blood flow measurements; E/A, ratio E-wave to A-wave by quantitative transmitral blood flow measurements; E, E-wave by quantitative transmitral blood flow measurements; A, A-wave by quantitative transmitral blood flow measurements; é, mean lateral and septal cine CMR velocities; E/é, ratio E-wave to é; MAPSE, Mitral annulus plain systolic excursion; SI, Sphericity index; BMI, body mass index; MM_i, BSA-indexed muscle-mass; $LV\text{-}EDV_i$, indexed left ventricular end diastolic volume; $LV\text{-}ESV_i$, indexed left ventricular end systolic volume; $LV\text{-}SV_i$, indexed left ventricular stroke volume.

IV. Correlations between left atrial *strain* measures and volumetric, hemodynamic and functional parameters – female subjects, total cohort

Female (N = 97)	**$LA\text{-}S_{res}$**	**$LA\text{-}S_{con}$**	**$LA\text{-}S_{boo}$**
**HR**	0.023	0.089	-0.105
**$LA\text{-}Vol_{max\_i}$**	-0.095	-0.191	0.140
**$LA\text{-}Vol_{min\_i}$**	-0.147	-0.250§	0.104
**$LA\text{-}Vol_{boo\_i}$**	-0.115	-0.303*	0.283*
**$LA\text{-}EF_{tot}$**	0.161	0.214§	0.024
**$LA\text{-}EF_{con}$**	0.115	0.340*	-0.322*
**$LA\text{-}EF_{boo}$**	0.125	-0.075	0.427*
**LV-EF**	0.235§	-0.268*	0.005
**LV-GLS**	0.177	0.220§	0.020
**LV-GCS**	0.273*	0.389*	-0.059
**LV-GRS**	-0.272*	-0.397*	0.063
**S/D**	-0.004	-0.181	0.333*
**E/A**	0.034	0.246§	-0.356*
**E**	0.125	0.253§	-0.209§
**A**	0.016	-0.154	0.269*
**E/e‘**	0.106	0.048	0.050
**e‘**	-0.051	0.062	-0.135
**MAPSE**	0.154	0.196	0.047
**SI**	-0.073	-0.011	-0.043
**BMI**	0.116	-0.037	0.324*
**$LV\text{-}EDV_i$**	0.024	0.129	-0.187
**$LV\text{-}ESV_i$**	0.214§	0.301*	-0.086
**$LV\text{-}SV_i$**	-0.140	-0.101	-0.128
**Age^+**	-0.146	-0.381*	0.364*
**MM_i^+**	-0.160	-0.117	-0.170

*Statistically significant ($p < 0.01$, two-sided).
§Statistically significant ($p < 0.05$, two-sided).

$LA\text{-}S_{res}$, reservoir left atrial strain; $LA\text{-}S_{con}$, conduit left atrial strain; $LA\text{-}S_{boo}$, booster left atrial strain; HR, heart rate; $LA\text{-}Vol_{max\_i}$, indexed maximum left atrial volume; $LA\text{-}Vol_{min\_i}$, indexed minimum left atrial volume; $LA\text{-}Vol_{boo\_i}$, indexed booster left atrial volume; $LA\text{-}EF_{total}$, total left atrial ejection fraction; $LA\text{-}EF_{con}$, passive left atrial ejection fraction; $LA\text{-}EF_{boo}$, booster left atrial ejection fraction; LV-EF, left ventricular ejection fraction; LV-GLS, global left ventricular longitudinal strain; LV-GCS, global left ventricular circumferential strain; LV-GRS, global left ventricular radial strain; S/D, ratio S-wave to D-wave by quantitative right upper pulmonary vein blood flow measurements; E/A, ratio E-wave to A-wave by quantitative transmitral blood flow measurements; E, E-wave by quantitative transmitral blood flow measurements; A, A-wave by quantitative transmitral blood flow measurements; é, mean lateral and septal cine CMR velocities; E/é, ratio E-wave to é; MAPSE, Mitral annulus plain systolic excursion; SI, Sphericity index; BMI, body mass index; MM_i, BSA-indexed muscle-mass; $LV\text{-}EDV_i$, indexed left ventricular end diastolic volume; $LV\text{-}ESV_i$, indexed left ventricular end systolic volume; $LV\text{-}SV_i$, indexed left ventricular stroke volume.

V. Correlations between left atrial *strain rates* and volumetric, hemodynamic and functional parameters – male subjects, total cohort

Male (N = 86)	$LA\text{-}SR_{res}$	$LA\text{-}SR_{con}$	$LA\text{-}SR_{boo}$
**HR**	0.329*	-0.378*	-0.456*
**$LA\text{-}Vol_{max\_i}$**	-0.305*	0.425*	0.198
**$LA\text{-}Vol_{min\_i}$**	-0.372*	0.428*	0.223§
**$LA\text{-}Vol_{boo\_i}$**	-0.328*	0.459*	0.171
**$LA\text{-}EF_{tot}$**	0.336*	-0.332*	-0.220§
**$LA\text{-}EF_{con}$**	0.233§	-0.330*	-0.036
**$LA\text{-}EF_{boo}$**	0.190	-0.048	-0.225§
**LV-EF**	0.121	-0.085	-0.222§
**LV-GLS**	-0.167	0.194	0.262§
**LV-GCS**	-0.222§	0.255§	0.354*
**LV-GRS**	0.235§	-0.254§	-0.370*
**S/D**	-0.188	0.263§	-0.167
**E/A**	0.085	-0.115	0.203
**E**	0.251§	-0.404*	-0.133
**A**	0.049	-0.094	-0.339*
**E/e'**	0.110	-0.027	-0.037
**e'**	0.017	-0.171	-0.031
**MAPSE**	0.115	-0.038	0.028
**SI**	0.029	0.043	0.040
**BMI**	-0.106	0.177	0.031
**$LV\text{-}EDV_i$**	-0.104	0.015	0.252§
**$LV\text{-}ESV_i$**	-0.159	0.058	0.329*
**$LV\text{-}SV_i$**	-0.021	-0.011	0.097
**Age**	-0.111	0.259§	-0.134
**MM_i**	-0.205	0.239§	0.226§

*Statistically significant ($p < 0.01$, two-sided).
§Statistically significant ($p < 0.05$, two-sided).
$LA\text{-}SR_{res}$, reservoir left atrial strain rate; $LA\text{-}SR_{con}$, conduit left atrial strain rate; $LA\text{-}SR_{boo}$, booster left atrial strain rate; HR, heart rate; $LA\text{-}Vol_{max\_i}$, indexed maximum left atrial volume; $LA\text{-}Vol_{min\_i}$, indexed minimum left atrial volume; $LA\text{-}Vol_{boo\_i}$, indexed booster left atrial volume; $LA\text{-}EF_{total}$, total left atrial ejection fraction; $LA\text{-}EF_{con}$, passive left atrial ejection fraction; $LA\text{-}EF_{boo}$, booster left atrial ejection fraction; LV-EF, left ventricular ejection fraction; LV-GLS, global left ventricular longitudinal strain; LV-GCS, global left ventricular circumferential strain; LV-GRS, global left ventricular radial strain; S/D, ratio S-wave to D-wave by quantitative right upper pulmonary vein blood flow measurements; E/A, ratio E-wave to A-wave by quantitative transmitral blood flow measurements; E, E-wave by quantitative transmitral blood flow measurements; A, A-wave by quantitative transmitral blood flow measurements; é, mean lateral and septal cine CMR velocities; E/é, ratio E-wave to é; MAPSE, Mitral annulus plain systolic excursion; SI, Sphericity index; BMI, body mass index; MM_i, BSA-indexed muscle-mass; $LV\text{-}EDV_i$, indexed left ventricular end diastolic volume; $LV\text{-}ESV_i$, indexed left ventricular end systolic volume; $LV\text{-}SV_i$, indexed left ventricular stroke volume.

VI. Correlations between left atrial *strain rates* and volumetric, hemodynamic and functional parameters – female subjects, total cohort

Female (N = 97)	LA-SR_{res}	LA-SR_{con}	LA-SR_{boo}
**HR**	0.153	-0.086	-0.299*
**LA-Vol_{max_i}**	-0.077	0.308*	0.007
**LA-Vol_{min_i}**	-0.124	-0.304*	0.034
**LA-Vol_{boo_i}**	-0.173	0.365*	-0.104
**LA-EF_{tot}**	0.158	-0.174	-0.048
**LA-EF_{con}**	0.206§	-0.290*	0.206§
**LA-EF_{boo}**	-0.016	0.079	-0.340*
**LV-EF**	-0.144	0.195	0.015
**LV-GLS**	0.199	-0.230§	0.054
**LV-GCS**	0.244§	-0.263*	0.120
**LV-GRS**	-0.253§	0.276*	-0.111
**S/D**	-0.058	0.181	-0.366*
**E/A**	0.074	-0.221§	0.379*
**E**	0.189	-0.243§	0.186
**A**	-0.003	0.096	-0.307*
**E/e'**	0.149	-0.102	-0.069
**e'**	-0.057	-0.015	0.136
**MAPSE**	0.111	-0.072	0.109
**SI**	0.027	0.044	-0.006
**BMI**	-0.018	0.067	-0.280*
**LV-EDV_i**	0.008	-0.025	0.310*
**LV-ESV_i**	0.127	-0.166	0.139
**LV-SV_i**	-0.130	0.136	0.270*
**Age**	-0.201§	0.368*	-0.316*
**MM_i**	-0.100	0.192	0.274*

*Statistically significant ($p < 0.01$, two-sided).
§Statistically significant ($p < 0.05$, two-sided).
LA-SR_{res}, reservoir left atrial strain rate; LA-SR_{con}, conduit left atrial strain rate; LA-SR_{boo}, booster left atrial strain rate; HR, heart rate; LA-Vol_{max_i}, indexed maximum left atrial volume; LA-Vol_{min_i}, indexed minimum left atrial volume; LA-Vol_{boo_i}, indexed booster left atrial volume; LA-EF_{total}, total left atrial ejection fraction; LA-EF_{con}, passive left atrial ejection fraction; LA-EF_{boo}, booster left atrial ejection fraction; LV-EF, left ventricular ejection fraction; LV-GLS, global left ventricular longitudinal strain; LV-GCS, global left ventricular circumferential strain; LV-GRS, global left ventricular radial strain; S/D, ratio S-wave to D-wave by quantitative right upper pulmonary vein blood flow measurements; E/A, ratio E-wave to A-wave by quantitative transmitral blood flow measurements; E, E-wave by quantitative transmitral blood flow measurements; A, A-wave by quantitative transmitral blood flow measurements; é, mean lateral and septal cine CMR velocities; E/é, ratio E-wave to é; MAPSE, Mitral annulus plain systolic excursion; SI, Sphericity index; BMI, body mass index; MM_i, BSA-indexed muscle-mass; LV-EDV_i, indexed left ventricular end diastolic volume; LV-ESV_i, indexed left ventricular end systolic volume; LV-SV_i, indexed left ventricular stroke volume.

Supplement S2

Table S2a: Multilinear regression analysis to study multifactorial influences of left atrial strain. Analysis was done on the total female study group (N = 97) using all heart rates.

	Validity*		Parameter	R	R^2	corr R^2	ANOVA significance	Sample size†	Goodness-of-fit	Function
reservoir	1	**$LA\text{-}S_{res}$**	$LA\text{-}Vol_{min\_i}$, $LA\text{-}EF_{tot}$, LV-GCS E	-	-	-	-	-	-	-
reservoir	2	**$LA\text{-}S_{res}$**	E, LV-GCS $LA\text{-}EF_{tot}$	0.398	0.158	0.131	0.001	80	medium	$LA\text{-}S_{res}$ = 0.659 * $LA\text{-}EF_{tot}$ + 1.946 * LV-GCS + 0.152 * E + 44.010
reservoir	3	**$LA\text{-}S_{res}$**	LV-GRS, LV-EF, $LA\text{-}EF_{tot}$, MMi	0.380	0.144	0.106	0.007	97	medium	$LA\text{-}S_{res}$ = -0.627 * LV-GRS - 0.066 * LV-EF + 0.548 * $LA\text{-}EF_{tot}$ – 0.285 * MM_i + 61.163
reservoir	3a	**$LA\text{-}S_{res}$**	LV-GRS $LA\text{-}EF_{tot}$	0.348	0.121	0.102	0.003	95	small	$LA\text{-}S_{res}$ = -0.606 * LV-GRS + 0.623 * $LA\text{-}EF_{tot}$ + 37.537

**conduit**	**4**	**LA-S$_{con}$**	LA-Vol$_{boo_i}$,<br>LA-EF$_{tot}$,<br>LV-GRS<br>E	0.515	0.265	0.233	< 0.001	48	large	LA-S$_{con}$ =<br>-0.144 * LA-V$_{boo_i}$ + 0.463 * LA-EF$_{tot}$ -<br>0.613 * LV-GRS + 0.270 * E + 18.627
	**5**	**LA-S$_{con}$**	LA-Vol$_{min_i}$,<br>LV-GCS,<br>E,<br>S/D	0.520	0.270	0.238	<0.001	47	large	LA-S$_{con}$ =<br>-0.656 * LA-V$_{min_i}$ + 2.233 * LV-GCS +<br>0.376 * E + 5.141 * S/D + 64.121
	**6**	**LA-S$_{con}$**	LA-EF$_{con}$,<br>LV-GCS,<br>age<br>LV-ESV$_i$	0.498	0.248	0.215	<0.001	52	medium	LA-S$_{con}$ =<br>0.308 * LA-EF$_{con}$ + 1.528 * LV-GCS -<br>0.160 * age - 0.137 * LV-ESV$_i$ + 61.851
	**6a**	**LA-S$_{con}$**	LV-GCS,<br>LA-Vol$_{min_i}$<br>E	0.500	0.250	0.226	<0.001	47	medium	LA-S$_{con}$ =<br>1.954 * LV-GCS - 0.503 * LA-V$_{min_i}$ +<br>0.316 * E + 64.522
**booster**	**7**	**LA-S$_{boo}$**	LA-EF$_{boo}$,<br>LV-EF,<br>A<br>S/D	0.569	0.324	0.293	< 0.001	38	large	LA-S$_{boo}$ =<br>0.282 * LA-EF$_{boo}$ - 0.293 * LV-EF - 0.022<br>* A + 4.194 * S/D + 23.432
	**8**	**LA-S$_{boo}$**	LA-EF$_{boo}$,<br>LV-ESV$_i$,<br>age<br>A	0.553	0.306	0.275	<0.001	41	large	LA-S$_{boo}$ =<br>0.315 * LA-EF$_{boo}$ + 0.307 * LV-ESVi +<br>0.100 * age + 0.008 * A – 4.055
	**9**	**LA-S$_{boo}$**	LA-Vol$_{boo_i}$,<br>LA-EF$_{boo}$,<br>MM$_i$<br>BMI	0.492	0.242	0.208	<0.001	54	medium	LA-S$_{boo}$ = 0.111 * LA-V$_{boo_i}$ + 0.217 * LA-<br>EF$_{boo}$ - 0.079 * MM$_i$ + 0.175 * BMI +<br>6.784
	**10**	**LA-S$_{boo}$**	LA-EF$_{boo}$,<br>LV-ESV$_i$<br>A	0.490	0.240	0.215	<0.001	49	medium	LA-S$_{boo}$ =<br>0.320 * LA-EF$_{boo}$ + 0.209 * LV-ESV$_i$ +<br>0.082 * A - 0.628

**reservoir**	**1**	**LA-SR$_{res}$**	LA_Vol$_{min_i}$,<br>LV-GRS;<br>E<br>age	0.340	0.116	0.076	0.024	**123**	small	LA-SR$_{res}$ =<br>-0.020 * LA_V$_{min_i}$ - 0.024 * LV-GRS +<br>0.012 * E - 0.005 * age + 3.131
	**2**	**LA-SR$_{res}$**	LA_Vol$_{min_i}$<br>E	0.256	0.066	0.046	0.042	**183**	small	LA-SR$_{res}$ =<br>-0.027 * LA_V$_{min_i}$ + 0.016 * E + 1.997
**conduit**	**3**	**LA-SR$_{con}$**	LA_Vol$_{boo_i}$,<br>LA-EF$_{tot}$,<br>S/D<br>E	0.448	0.201	0.165	0.001	67	medium	LA-SR$_{con}$ =<br>0.069 * LA_V$_{boo_i}$ - 0.007 * LA-EF$_{tot}$ -<br>0.467 * S/D - 0.039 * E – 2.360
	**4**	**LA-SR$_{con}$**	LA-EF$_{con}$,<br>LV-GRS,<br>MM$_i$<br>age	0.540	0.292	0.259	<0.001	43	large	LA-SR$_{con}$ =<br>-0.035 * LA-EF$_{con}$ + 0.048 * LV-GRS +<br>0.057 * MM$_i$ + 0.016 * age – 7.405
	**5**	**LA-SR$_{con}$**	LA_Vol$_{boo_i}$<br>E	0.438	0.192	0.174	<0.001	57	medium	LA-SR$_{con}$ =<br>0.059 * LA_V$_{boo_i}$ - 0.037* E – 3.099
**booster**	**6**	**LA-SR$_{boo}$**	LA-EF$_{boo}$,<br>LV-GCS,<br>S/D<br>A	0.441	0.195	0.159	0.001	69	medium	LA-SR$_{boo}$ =<br>-0.030 * LA-EF$_{boo}$ - 0.021 * LV-GCS -<br>0.488 * S/D - 0.016 * A – 0.875
	**7**	**LA-SR$_{boo}$**	LA-EF$_{boo}$,<br>A,<br>LV-EDV$_i$<br>MM$_i$	0.441	0.195	0.159	0.001	69	medium	LA-SR$_{boo}$ =<br>-0.017 * LA-EF$_{boo}$ - 0.026 *A - 0.004 *<br>LV-EDV$_i$ + 0.036 * MM$_i$ – 2.626

Note: In order to be able to carry out a multilinear regression analysis, it is mandatory to check the prerequisites in advance. These are (a) linear relationships between the variables, (b) no outliers, (c) independence of the residuals, (d) no multicollinearity, (e) homoscedasticity and (f) normal distribution.

[*] green = prerequisites are met; orange = valid, but with fewer concerns due to the prerequisites (e.g. an outlier had to be removed or the independence of the residuals had not reached the optimal value of ~2 before the analysis); red = not valid due to violation of prerequisites.

Interpretation of |R²| according to Cohen. [Cohen, J. (1988). Statistical power analysis for the behavioral sciences (2nd ed.). Hillsdale, N.J.: L. Erlbaum Associates. Page 412 ff].
small effect size: $|R^2| = .02$; medium effect size $|R^2| = .13$; large effect size $|R^2| = .26$.
[†] Sample size calculation see reference [Calculation according to Hemmerich, W. (2019). StatistikGuru: Poweranalyse und Stichprobenberechnung für Regression. Retrieved from https://statistikguru.de/rechner/poweranalyse-regression.html].
LA-S_{res}, reservoir left atrial strain; LA-S_{con}, conduit left atrial strain; LA-S_{boo}, booster left atrial strain; LA-SR_{res}, reservoir left atrial strain rate; LA-SR_{con}, conduit left atrial strain rate; LA-SR_{boo}, booster left atrial strain rate; LA-Vol_{max_i}, indexed maximum left atrial volume; LA-Vol_{min_i}, indexed minimum left atrial volume; LA-Vol_{boo_i}, indexed booster left atrial volume; LA-EF_{total}, total left atrial ejection fraction; LA-EF_{con}, passive left atrial ejection fraction; LA-EF_{boo}, booster left atrial ejection fraction; LV-EF, left ventricular ejection fraction; LV-GLS, global left ventricular longitudinal strain; LV-GCS, global left ventricular circumferential strain; LV-GRS, global left ventricular radial strain; S/D, ratio S-wave to D-wave by quantitative right upper pulmonary vein blood flow measurements; E/A, ratio E-wave to A-wave by quantitative transmitral blood flow measurements; E, E-wave by quantitative transmitral blood flow measurements; A, A-wave by quantitative transmitral blood flow measurements; é, mean lateral and septal cine CMR velocities; E/é, ratio E-wave to é; MAPSE, Mitral annulus plain systolic excursion; SI, Sphericity index; MM_i, BSA-indexed muscle-mass; LV-EDV_i, indexed left ventricular end diastolic volume; LV-ESV_i, indexed left ventricular end systolic volume; LV-SV_i, indexed left ventricular stroke volume.

Table S2b: Multilinear regression analysis to study multifactorial influences of left atrial strain. Analysis was done on the total male study group (N = 86) using all heart rates.

	validity		Parameter	R	R^2	corr R^2	ANOVA significance	Sample size†	Goodness-of-fit	Function
reservoir	1	**LA-S_{res}**	LA-Vol_{min_i}, LA-EF_{tot}, LV-GCS E	0.463	0.215	0.172	<0.001	62	medium	LA-S_{res} = -0.262* LA-V_{min_i} + 0.302* LA-EF_{tot} – 0.842* LV-GCS + 0.182*E + 7.836
	2	**LA-S_{res}**	E, LV-GCS LA-EF_{tot}	0.452	0.205	0.172	0.001	60	medium	LA-S_{res} = 0.521* LA-EF_{tot} – 0.891* LV-GCS + 0.187 * E – 11.379
	3	**LA-S_{res}**	LV-GRS, LV-EF, LA-EF_{tot}, MM_i	0.428	0.183	0.138	0.005	74	medium	LA-S_{res} = 0.273 * LV-GRS + 0.100 * LV-EF + 0.594 * LA-EF_{tot} – 0.088 * MM_i + 1.172

**conduit**	**4**	**LA-S$_{con}$**	LA-Vol$_{boo_i}$,<br>LA-EF$_{tot}$,<br>LV-GRS<br>E	0.474	0.225	0.181	0.001	59	medium	LA-S$_{con}$ =<br>-0.124 * LA-V$_{boo_i}$ + 0.418 * LA-EF$_{tot}$ +<br>0.064 * LV-GRS + 0.175 * E – 2.450
	**5**	**LA-S$_{con}$**	LA-Vol$_{min_i}$,<br>LV-GCS,<br>E,<br>S/D	0.451	0.203	0.160	0.002	66	medium	LA-S$_{con}$ =<br>-0.383 * LA-V$_{min_i}$ - 0.404 * LV-GCS +<br>0.204 * E - 0.535 * S/D + 18.604
	**6**	**LA-S$_{con}$**	LA-EF$_{con}$,<br>LV-GCS,<br>age<br>LV-ESV$_i$	0.415	0.173	0.127	0.008	79	medium	LA-S$_{con}$ =<br>0.470 * LA-EF$_{con}$ - 0.771 * LV-GCS +<br>0.026 * age + 0.046 * LV-ESV$_i$ – 3.145
**booster**	**7**	**LA-S$_{boo}$**	LA-EF$_{boo}$,<br>LV-EF,<br>A<br>S/D	0.395	0.156	0.111	0.011	89	medium	LA-S$_{boo}$ =<br>0.156 * LA-EF$_{boo}$ + 0.195 * LV-EF +<br>0.044 * A + 1.908 * S/D – 5.230
	**8**	**LA-S$_{boo}$**	LA-EF$_{boo}$,<br>LV-ESV$_i$,<br>age<br>A	0.447	0.200	0.156	0.002	67	medium	LA-S$_{boo}$ =<br>0.106 * LA-EF$_{boo}$ - 0.292 * LV-ESV$_i$ +<br>0.025 * age + 0.039 * A + 18.547
	**9**	**LA-S$_{boo}$**	LA-Vol$_{boo_i}$,<br>LA-EF$_{boo}$,<br>MM$_i$<br>BMI	0.341	0.116	0.069	0.054	**123**	small	LA-S$_{boo}$ =<br>0.061 * LA-V$_{boo_i}$ + 0.180 * LA-EF$_{boo}$ -<br>0.158 * MM$_i$ - 0.139 * BMI + 21.047
	**10**	**LA-S$_{boo}$**	LA-EF$_{boo}$,<br>LV-ESV$_i$<br>A	0.442	0.195	0.163	0.001	63	medium	LA-S$_{boo}$ =<br>0.111 * LA-EF$_{boo}$ - 0.296 * LV-ESV$_i$ +<br>0.066 * A + 18.592

**reservoir**	**1**	**LA-SR$_{res}$**	LA_Vol$_{min_i}$,<br>LV-GRS;<br>E<br>age	0.557	0.310	0.271	<0.001	40	large	LA-SR$_{res}$ =<br>-0.051 * LA_V$_{min_i}$ + 0.011 * LV-GRS +<br>0.016 * E + 0.008 * age + 1.786
	**2**	**LA-SR$_{res}$**	LA_Vol$_{min_i}$<br>E	0.514	0.265	0.246	<0.001	39	large	LA-SR$_{res}$ =<br>-0.043 * LA_V$_{min_i}$ + 0.011 * E + 2.505
**conduit**	**3**	**LA-SR$_{con}$**	LA_Vol$_{boo_i}$,<br>LA-EF$_{tot}$,<br>S/D<br>E	0.540	0.291	0.252	<0.001	43	large	LA-SR$_{con}$ =<br>0.034 * LA_V$_{boo_i}$ – 7*E-5 * LA-EF$_{tot}$ +<br>0.028 * S/D - 0.038* E – 2.142
	**4**	**LA-SR$_{con}$**	LA-EF$_{con}$,<br>LV-GRS,<br>MM$_i$<br>age	0.502	0.252	0.210	<0.001	51	medium	LA-SR$_{con}$ =<br>-0.048* LA-EF$_{con}$ - 0.045* LV-GRS +<br>0.004* MM$_i$ + 0.007* age – 0.583
	**5**	**LA-SR$_{con}$**	LA_Vol$_{boo_i}$<br>E	0.542	0.293	0.275	<0.001	34	large	LA-SR$_{con}$ =<br>0.035 * LA_V$_{boo_i}$ - 0.039* E – 2.104
**booster**	**6**	**LA-SR$_{boo}$**	LA-EF$_{boo}$,<br>LV-GCS,<br>S/D<br>A	0.440	0.194	0.148	0.004	69	medium	LA-SR$_{boo}$ =<br>-0.016 * LA-EF$_{boo}$ + 0.133 * LV-GCS +<br>0.233 * S/D - 0.034 * A + 1.503
	**7**	**LA-SR$_{boo}$**	LA-EF$_{boo}$,<br>A,<br>LV-EDV$_i$<br>MM$_i$	0.414	0.172	0.127	0.007	86	medium	LA-SR$_{boo}$ =<br>-0.019 * LA-EF$_{boo}$ - 0.032 *A + 0.011 *<br>LV-EDV$_i$ + 0.015 * MM$_i$ – 2.387

Note: In order to be able to carry out a multilinear regression analysis, it is mandatory to check the prerequisites in advance. These are (a) linear relationships between the variables, (b) no outliers, (c) independence of the residuals, (d) no multicollinearity, (e) homoscedasticity and (f) normal distribution.

[*] green = prerequisites are met; orange = valid, but with fewer concerns due to the prerequisites (e.g. an outlier had to be removed or the independence of the residuals had not reached the optimal value of ~2 before the analysis); red = not valid due to violation of prerequisites.

Interpretation of |R^2| according to Cohen. [Cohen, J. (1988). Statistical power analysis for the behavioral sciences (2nd ed.). Hillsdale, N.J.: L. Erlbaum Associates. Page 412 ff].
small effect size:|R^2| = .02; medium effect size |R^2| = .13; large effect size |R^2| = .26.
[†] Sample size calculation see reference [Calculation according to Hemmerich, W. (2019). StatistikGuru: Poweranalyse und Stichprobenberechnung für Regression. Retrieved from https://statistikguru.de/rechner/poweranalyse-regression.html].
LA-S_{res}, reservoir left atrial strain; LA-S_{con}, conduit left atrial strain; LA-S_{boo}, booster left atrial strain; LA-SR_{res}, reservoir left atrial strain rate; LA-SR_{con}, conduit left atrial strain rate; LA-SR_{boo}, booster left atrial strain rate; LA-Vol_{max_i}, indexed maximum left atrial volume; LA-Vol_{min_i}, indexed minimum left atrial volume; LA-Vol_{boo_i}, indexed booster left atrial volume; LA-EF_{total}, total left atrial ejection fraction; LA-EF_{con}, passive left atrial ejection fraction; LA-EF_{boo}, booster left atrial ejection fraction; LV-EF, left ventricular ejection fraction; LV-GLS, global left ventricular longitudinal strain; LV-GCS, global left ventricular circumferential strain; LV-GRS, global left ventricular radial strain; S/D, ratio S-wave to D-wave by quantitative right upper pulmonary vein blood flow measurements; E/A, ratio E-wave to A-wave by quantitative transmitral blood flow measurements; E, E-wave by quantitative transmitral blood flow measurements; A, A-wave by quantitative transmitral blood flow measurements; é, mean lateral and septal cine CMR velocities; E/é, ratio E-wave to é; MAPSE, Mitral annulus plain systolic excursion; SI, Sphericity index; MM_i, BSA-indexed muscle-mass; LV-EDV_i, indexed left ventricular end diastolic volume; LV-ESV_i, indexed left ventricular end systolic volume; LV-SV_i, indexed left ventricular stroke volume.

Table S2c: Multi-linear regression analysis to study multifactorial influences of left atrial strain. Analysis was done on female subjects (N = 60) with heart rates between 60 – 75 bpm.

	validity		Parameter	R	R^2	corr R^2	ANOVA significance	Sample size†	Goodness-of-fit	Function
reservoir	1	**$LA\text{-}S_{res}$**	$LA\text{-}Vol_{min\_i}$, $LA\text{-}EF_{tot}$, LV-GCS E	-	-	-	-	-	-	-
	2	**$LA\text{-}S_{res}$**	E, LV-GCS $LA\text{-}EF_{tot}$	0.527	0.278	0.238	< 0.001	41	large	$LA\text{-}S_{res}$ = 0.825 * $LA\text{-}EF_{tot}$ + 2.633 * LV-GCS + 0.250 * E + 44.270
	3	**$LA\text{-}S_{res}$**	LV-GRS, LV-EF, $LA\text{-}EF_{tot}$, MM_i	0.459	0.211	0.151	0.012	63	medium	$LA\text{-}S_{res}$ = -0.846 * LV-GRS + 0.227 * LV-EF + 0.807 * $LA\text{-}EF_{tot}$ - 0.159 * MM_i + 29.681
	3a	**$LA\text{-}S_{res}$**	LV-GRS $LA\text{-}EF_{tot}$	0.451	0.204	0.175	0.002	53	medium	$LA\text{-}S_{res}$ = -0.747 * LV-GRS + 0.839 * $LA\text{-}EF_{tot}$ + 31.363

**conduit**	**4**	**LA-S$_{con}$**	LA-Vol$_{boo_i}$,<br>LA-EF$_{tot}$,<br>LV-GRS<br>E	0.661	0.437	0.395	< 0.001	26	large	LA-S$_{con}$ =<br>0.185 * LA-V$_{boo_i}$ + 0.931 * LA-EF$_{tot}$ –<br>1.056 * LV-GRS + 0.351 * E - 5.403
	**5**	**LA-S$_{con}$**	LA-Vol$_{min_i}$,<br>LV-GCS,<br>E,<br>S/D	0.632	0.400	0.355	<0.001	29	large	LA-S$_{con}$ =<br>-0.520 * LA-V$_{min_i}$ + 3.227 * LV-GCS +<br>0.493 * E + 5.220 * S/D + 75.137
	**6**	**LA-S$_{con}$**	LA-EF$_{con}$,<br>LV-GCS,<br>age<br>LV-ESV$_{i}$	0.640	0.410	0.366	<0.001	28	large	LA-S$_{con}$ =<br>0.651 * LA-EF$_{con}$ + 1.774 * LV-GCS -<br>0.080 * age + 0.207 * LV-ESV$_{i}$ + 45.528
	**6a**	**LA-S$_{con}$**	LV-GCS,<br>LA-Vol$_{min_i}$<br>E	0.607	0.369	0.334	<0.001	29	large	LA-S$_{con}$ =<br>2.970 * LV-GCS - 0.183 * LA-V$_{min_i}$ +<br>0.454 * E + 71.372
**booster**	**7**	**LA-S$_{boo}$**	LA-EF$_{boo}$,<br>LV-EF,<br>A<br>S/D	0.635	0.404	0.359	< 0.001	28	large	LA-S$_{boo}$ =<br>0.405 * LA-EF$_{boo}$ - 0.278 * LV-EF + 0.280<br>* A + 2.107 * S/D + 12.264
	**8**	**LA-S$_{boo}$**	LA-EF$_{boo}$,<br>LV-ESV$_{i}$,<br>age<br>A	0.647	0.418	0.374	<0.001	27	large	LA-S$_{boo}$ =<br>0.430 * LA-EF$_{boo}$ + 0.286 * LV-ESV$_{i}$ +<br>0.116 * age + 0.138 * A – 11.988
	**9**	**LA-S$_{boo}$**	LA-Vol$_{boo_i}$,<br>LA-EF$_{boo}$,<br>MM$_{i}$<br>BMI	0.564	0.318	0.267	<0.001	39	large	LA-S$_{boo}$ =<br>0.233 * LA-V$_{boo_i}$ + 0.397 * LA-EF$_{boo}$ -<br>0.156 * MM$_{i}$ + 0.041 * BMI + 3.628
	**10**	**LA-S$_{boo}$**	LA-EF$_{boo}$,<br>LV-ESV$_{i}$<br>A	0.611	0.373	0.339	<0.001	29	large	LA-S$_{boo}$ =<br>0.428 * LA-EF$_{boo}$ + 0.152 * LV-ESV$_{i}$ +<br>0.317 * A – 9.311

**reservoir**	**1**	**LA-SR$_{res}$**	LA_Vol$_{min_i}$,<br>LV-GRS;<br>E<br>age	0.456	0.208	0.147	0.015	**64**	medium	LA-SR$_{res}$ =<br>-0.018 * LA_V$_{min_i}$ - 0.036 * LV-GRS +<br>0.019 * E - 0.003 * age + 2.956
	**2**	**LA-SR$_{res}$**	LA_Vol$_{min_i}$<br>E	0.334	0.111	0.080	0.037	**105**	small	LA-SR$_{res}$ =<br>-0.034 * LA_V$_{min_i}$ + 0.018 * E + 1.962
**conduit**	**3**	**LA-SR$_{con}$**	LA_Vol$_{boo_i}$,<br>LA-EF$_{tot}$,<br>S/D<br>E	0.593	0.351	0.301	<0.001	34	large	LA-SR$_{con}$ =<br>0.056 * LA_V$_{boo_i}$ - 0.039 * LA-EF$_{tot}$ -<br>0.054 * S/D - 0.041 * E – 0.615
	**4**	**LA-SR$_{con}$**	LA-EF$_{con}$,<br>LV-GRS,<br>MM$_i$<br>age	0.586	0.344	0.293	<0.001	35	large	LA-SR$_{con}$ =<br>-0.049 * LA-EF$_{con}$ + 0.068 * LV-GRS +<br>0.042 * MM$_i$ + 0.013 * age – 6.823
	**5**	**LA-SR$_{con}$**	LA_Vol$_{boo_i}$<br>E	0.579	0.335	0.310	<0.001	29	large	LA-SR$_{con}$ =<br>0.067 * LA_V$_{boo_i}$ - 0.043 * E – 3.056
**booster**	**6**	**LA-SR$_{boo}$**	LA-EF$_{boo}$,<br>LV-GCS,<br>S/D<br>A	0.432	0.187	0.125	0.027	**72**	medium	LA-SR$_{boo}$ =<br>-0.015 * LA-EF$_{boo}$ - 0.041 * LV-GCS -<br>0.477 * S/D - 0.014 * A – 1.693
	**7**	**LA-SR$_{boo}$**	LA-EF$_{boo}$,<br>A,<br>LV-EDV$_i$<br>MM$_i$	0.505	0.255	0.199	0.003	51	medium	LA-SR$_{boo}$ =<br>-0.008 * LA-EF$_{boo}$ - 0.035 *A - 0.019 *<br>LV-EDV$_i$ + 0.041 * MM$_i$ - 1.773
	**7a**	**LA-SR$_{boo}$**	A,<br>MM$_i$	0.466	0.217	0.189	0.001	49	medium	LA-SR$_{boo}$ =<br>-0.030 * A + 0.032 * MM$_i$ – 3.085

Note: In order to be able to carry out a multilinear regression analysis, it is mandatory to check the prerequisites in advance. These are (a) linear relationships between the variables, (b) no outliers, (c) independence of the residuals, (d) no multicollinearity, (e) homoscedasticity and (f) normal distribution.

[*] green = prerequisites are met; orange = valid, but with fewer concerns due to the prerequisites (e.g. an outlier had to be removed or the independence of the residuals had not reached the optimal value of ~2 before the analysis); red = not valid due to violation of prerequisites.

Interpretation of $|R^2|$ according to Cohen. [Cohen, J. (1988). Statistical power analysis for the behavioral sciences (2nd ed.). Hillsdale, N.J.: L. Erlbaum Associates. Page 412 ff].

small effect size: $|R^2| = .02$; medium effect size $|R^2| = .13$; large effect size $|R^2| = .26$.

[†] Sample size calculation see reference [Calculation according to Hemmerich, W. (2019). StatistikGuru: Poweranalyse und Stichprobenberechnung für Regression. Retrieved from https://statistikguru.de/rechner/poweranalyse-regression.html].

LA-S_{res}, reservoir left atrial strain; LA-S_{con}, conduit left atrial strain; LA-S_{boo}, booster left atrial strain; LA-SR_{res}, reservoir left atrial strain rate; LA-SR_{con}, conduit left atrial strain rate; LA-SR_{boo}, booster left atrial strain rate; LA-Vol_{max_i}, indexed maximum left atrial volume; LA-Vol_{min_i}, indexed minimum left atrial volume; LA-Vol_{boo_i}, indexed booster left atrial volume; LA-EF_{total}, total left atrial ejection fraction; LA-EF_{con}, passive left atrial ejection fraction; LA-EF_{boo}, booster left atrial ejection fraction; LV-EF, left ventricular ejection fraction; LV-GLS, global left ventricular longitudinal strain; LV-GCS, global left ventricular circumferential strain; LV-GRS, global left ventricular radial strain; S/D, ratio S-wave to D-wave by quantitative right upper pulmonary vein blood flow measurements; E/A, ratio E-wave to A-wave by quantitative transmitral blood flow measurements; E, E-wave by quantitative transmitral blood flow measurements; A, A-wave by quantitative transmitral blood flow measurements; é, mean lateral and septal cine CMR velocities; E/é, ratio E to é; MAPSE, Mitral annulus plain systolic excursion; SI, Sphericity index; MM_i, BSA-indexed muscle-mass; LV-EDV_i, indexed left ventricular end diastolic volume; LV-ESV_i, indexed left ventricular end systolic volume; LV-SV_i, indexed left ventricular stroke volume.

Table S2d: Multi-linear regression analysis to study multifactorial influences of left atrial strain. Analysis was done on male subjects (N = 46) with heart rates between 60 – 75 bpm.

	validity		Parameter	R	R^2	corr R^2	ANOVA significance	Sample size†	Goodness-of-fit	Function
reservoir	1	$LA\text{-}S_{res}$	$LA\text{-}Vol_{min\_i}$, $LA\text{-}EF_{tot}$, LV-GCS E	-	-	-	-	-	-	-
	2	$LA\text{-}S_{res}$	E, LV-GCS $LA\text{-}EF_{tot}$	0.565	0.320	0.267	0.002	35	large	$LA\text{-}S_{res}$ = 0.897 * $LA\text{-}EF_{tot}$ - 1.114 * LV-GCS + 0.212 * E – 36.764
	3	$LA\text{-}S_{res}$	LV-GRS, LV-EF, $LA\text{-}EF_{tot}$, MM_i	0.502	0.252	0.173	0.023	**51**	medium	$LA\text{-}S_{res}$ = 0.455 * LV-GRS - 0.122 * LV-EF + 0.898 * $LA\text{-}EF_{tot}$ – 0.030 * MM_i - 10.139

**conduit**	**4**	**LA-S_{con}**	LA-Vol_{boo_i},<br>LA-EF_{tot},<br>LV-GRS<br>E	0.637	0.405	0.341	0.001	28	large	LA-S_{con} =<br>-0.004 * LA-V_{boo_i} + 0.458 * LA-EF_{tot} +<br>0.030 * LV-GRS + 0.437 * E - 21.650
	**5**	**LA-S_{con}**	LA-Vol_{min_i},<br>LV-GCS,<br>E,<br>S/D	0.628	0.395	0.327	0.001	29	large	LA-S_{con} =<br>-0.528 * LA-V_{min_i} + 0.508 * LV-GCS +<br>0.589 * E + 4.312 * S/D + 12.336
	**6**	**LA-S_{con}**	LA-EF_{con},<br>LV-GCS,<br>age<br>LV-ESV_i	0.488	0.238	0.160	0.028	55	medium	LA-S_{con} =<br>0.764 * LA-EF_{con} - 0.438 * LV-GCS +<br>0.167 * age - 0.046 * LV-ESV_i – 7.651
**booster**	**7**	**LA-S_{boo}**	LA-EF_{boo},<br>LV-EF,<br>A<br>S/D	0.427	0.182	0.100	0.083	75	medium	LA-S_{boo} =<br>0.254 * LA-EF_{boo} + 0.133 * LV-EF +<br>0.175 * A - 1.699 * S/D – 4.050
	**8**	**LA-S_{boo}**	LA-EF_{boo},<br>LV-ESV_i,<br>age<br>A	0.466	0.217	0.139	0.040	61	medium	LA-S_{boo} =<br>0.209 * LA-EF_{boo} - 0.259 * LV-ESV_i -<br>0.008 * age + 0.120 * A + 13.311
	**9**	**LA-S_{boo}**	LA-Vol_{boo_i},<br>LA-EF_{boo},<br>MM_i<br>BMI	0.412	0.170	0.087	0.106	81	medium	LA-S_{boo} =<br>0.009 * LA-V_{boo_i} + 0.249 * LA-EF_{boo} -<br>0.157 * MM_i - 0.110 * BMI + 20.295
	**10**	**LA-S_{boo}**	LA-EF_{boo},<br>LV-ESV_i<br>A	0.465	0.217	0.159	0.018	56	medium	LA-S_{boo} =<br>0.205 * LA-EF_{boo} - 0.255 * LV-ESV_i +<br>0.110 * A + 13.322

**reservoir**	**1**	**LA-SR$_{res}$**	LA_Vol$_{min_i}$,<br>LV-GRS;<br>E<br>age	0.619	0.383	0.316	0.001	31	large	LA-SR$_{res}$ =<br>-0.074 * LA_V$_{min_i}$ + 0.009 * LV-GRS +<br>0.023 * E + 0.014 * age + 1.747
	**2**	**LA-SR$_{res}$**	LA_Vol$_{min_i}$<br>E	0.479	0.229	0.191	0.005	46	medium	LA-SR$_{res}$ =<br>-0.046 * LA_V$_{min_i}$ + 0.016 * E + 2.242
**conduit**	**3**	**LA-SR$_{con}$**	LA_Vol$_{boo_i}$,<br>LA-EF$_{tot}$,<br>S/D<br>E	0.551	0.304	0.230	0.007	41	large	LA-SR$_{con}$ =<br>0.027 * LA_V$_{boo_i}$ + 0.018 * LA-EF$_{tot}$ -<br>0.119 * S/D - 0.056 * E – 1.774
	**4**	**LA-SR$_{con}$**	LA-EF$_{con}$,<br>LV-GRS,<br>MM$_i$<br>age	0.580	0.337	0.265	0.004	36	large	LA-SR$_{con}$ =<br>-0.105 * LA-EF$_{con}$ - 0.027 * LV-GRS -<br>0.025 * MM$_i$ - 0.021 * age + 3.445
	**5**	**LA-SR$_{con}$**	LA_Vol$_{boo_i}$<br>E	0.567	0.321	0.288	<0.001	30	large	LA-SR$_{con}$ =<br>0.019 * LA_V$_{boo_i}$ - 0.054 * E – 0.749
**booster**	**6**	**LA-SR$_{boo}$**	LA-EF$_{boo}$,<br>LV-GCS,<br>S/D<br>A	0.582	0.339	0.267	0.003	36	large	LA-SR$_{boo}$ =<br>-0.029 * LA-EF$_{boo}$ + 0.075 * LV-GCS +<br>0.029 * S/D - 0.032 * A + 1.023
	**7**	**LA-SR$_{boo}$**	LA-EF$_{boo}$,<br>A,<br>LV-EDV$_i$<br>MM$_i$	0.704	0.496	0.443	<0.001	22	large	LA-SR$_{boo}$ =<br>-0.020 * LA-EF$_{boo}$ - 0.035 *A + 0.027 *<br>LV-EDV$_i$ + 0.016 * MM$_i$ - 3.764

Note: In order to be able to carry out a multilinear regression analysis, it is mandatory to check the prerequisites in advance. These are (a) linear relationships between the variables, (b) no outliers, (c) independence of the residuals, (d) no multicollinearity, (e) homoscedasticity and (f) normal distribution.

[*] green = prerequisites are met; orange = valid, but with fewer concerns due to the prerequisites (e.g. an outlier had to be removed or the independence of the residuals had not reached the optimal value of ~2 before the analysis); red = not valid due to violation of prerequisites.

Interpretation of $|R^2|$ according to Cohen. [Cohen, J. (1988). Statistical power analysis for the behavioral sciences (2nd ed.). Hillsdale, N.J.: L. Erlbaum Associates. Page 412 ff].

small effect size:|R^2| = .02; medium effect size |R^2| = .13; large effect size |R^2| = .26.

† Sample size calculation see reference [Calculation according to Hemmerich, W. (2019). StatistikGuru: Poweranalyse und Stichprobenberechnung für Regression. Retrieved from https://statistikguru.de/rechner/poweranalyse-regression.html].

LA-S_{res}, reservoir left atrial strain; LA-S_{con}, conduit left atrial strain; LA-S_{boo}, booster left atrial strain; LA-SR_{res}, reservoir left atrial strain rate; LA-SR_{con}, conduit left atrial strain rate; LA-SR_{boo}, booster left atrial strain rate; LA-Vol_{max_i}, indexed maximum left atrial volume; LA-Vol_{min_i}, indexed minimum left atrial volume; LA-Vol_{boo_i}, indexed booster left atrial volume; LA-EF_{total}, total left atrial ejection fraction; LA-EF_{con}, passive left atrial ejection fraction; LA-EF_{boo}, booster left atrial ejection fraction; LV-EF, left ventricular ejection fraction; LV-GLS, global left ventricular longitudinal strain; LV-GCS, global left ventricular circumferential strain; LV-GRS, global left ventricular radial strain; S/D, ratio S-wave to D-wave by quantitative right upper pulmonary vein blood flow measurements; E/A, ratio E-wave to A-wave by quantitative transmitral blood flow measurements; E, E-wave by quantitative transmitral blood flow measurements; A, A-wave by quantitative transmitral blood flow measurements; é, mean lateral and septal cine CMR velocities; E/é, ratio E-wave to é; MAPSE, Mitral annulus plain systolic excursion; SI, Sphericity index; MM_i, BSA-indexed muscle-mass; LV-EDV_i, indexed left ventricular end diastolic volume; LV-ESV_i, indexed left ventricular end systolic volume; LV-SV_i, indexed left ventricular stroke volume.
